# Antibodies Targeting the PfRH1 Binding Domain Inhibit Invasion of *Plasmodium falciparum* Merozoites

**DOI:** 10.1371/journal.ppat.1000104

**Published:** 2008-07-11

**Authors:** Xiaohong Gao, Kim Pin Yeo, Siqi Sharon Aw, Claudia Kuss, Jayasree K. Iyer, Saraswathy Genesan, Ravikumar Rajamanonmani, Julien Lescar, Zbynek Bozdech, Peter R. Preiser

**Affiliations:** 1 Division of Genomics & Genetics, School of Biological Sciences, Nanyang Technological University, Singapore; 2 Division of Structural & Computational Biology, School of Biological Sciences, Nanyang Technological University, Singapore; Northwestern University Medical School, United States of America

## Abstract

Invasion by the malaria merozoite depends on recognition of specific erythrocyte surface receptors by parasite ligands. *Plasmodium falciparum* uses multiple ligands, including at least two gene families, reticulocyte binding protein homologues (RBLs) and erythrocyte binding proteins/ligands (EBLs). The combination of different RBLs and EBLs expressed in a merozoite defines the invasion pathway utilized and could also play a role in parasite virulence. The binding regions of EBLs lie in a conserved cysteine-rich domain while the binding domain of RBL is still not well characterized. Here, we identify the erythrocyte binding region of the *P. falciparum* reticulocyte binding protein homologue 1 (PfRH1) and show that antibodies raised against the functional binding region efficiently inhibit invasion. In addition, we directly demonstrate that changes in the expression of RBLs can constitute an immune evasion mechanism of the malaria merozoite.

## Author Summary

*Plasmodium falciparum* causes the most virulent form of human malaria. The pathology of the disease is associated with the invasion, replication and subsequent destruction of the erythrocyte by the parasite. Invasion of the host erythrocyte by the invasive form of the parasite, the merozoite, is a key step involving the interaction of several parasite ligands with receptors on the host cell surface. A better understanding of the molecular basis for these interactions is crucial for developing effective strategies to reduce morbidity and mortality due to malaria. Members of the RBLs and EBLs are found in all *Plasmodium spp.* so far analyzed and play an important role in parasite virulence, host cell selection and possibly immune evasion. How binding of EBLs or RBLs to specific erythrocyte receptors ultimately leads to merozoite invasion is an important question that requires the parasite ligand to be dissected into functional domains. Here, we show that a relatively small region of the PfRH1 molecule is involved in receptor recognition. Only parasites that utilize a sialic acid–dependent invasion pathway are inhibited by antiserum raised against the minimal binding region. In addition, switching of the invasion pathway from a sialic acid–dependent to a sialic acid–independent pathway renders the inhibitory antibodies ineffective with a concomitant reduction in the amount of PfRH1 expressed. This demonstrates that invasion pathway switching in *P. falciparum* can also serve as a mechanism of immune evasion.

## Introduction

Malaria is caused by parasites of the genus *Plasmodium* with an estimated 300–500 million clinical cases and 1–3 million deaths annually [1],[2]. *Plasmodium falciparum* is the most prevalent and is responsible for a large proportion of the mortality associated with this disease. An essential step in the life cycle of malaria parasites is the invasion of host erythrocytes by merozoites and this is also an ideal target for a vaccine based intervention strategy. The invasion process is characterized by a multitude of specific, but relatively poorly understood, interactions between protein ligands expressed by the merozoite and receptors at the erythrocyte surface [3]–[5]. A better understanding of the molecular basis for these interactions is crucial for developing effective strategies to reduce morbidity and mortality due to malaria. Several molecules implicated in the invasion process have been identified in the apical organelles (rhoptry, micronemes, and dense granules) of the merozoite. At least two gene families: the reticulocyte binding protein homologues (RBLs) and the family of erythrocyte binding proteins/ligands (EBLs), mediate specific interactions with host cell receptors thereby defining host cell tropism [4].

Members of the RBLs and EBLs are found in all *Plasmodium spp.* so far analyzed and play an important role in parasite virulence, host cell selection and possibly immune evasion [6],[7]. The number of RBLs present in different *Plasmodium spp.* varies from two reticulocyte binding proteins (RBP 1&2) identified in *P. vivax* to 14 copies of the 235 kDa rhoptry protein (PY235) seen in the rodent malaria parasite *P. yoelii* [7]. In *P. falciparum* six RBL members have been identified, PfRH1 [8], PfRH2a & 2b [9],[10], PfRH3, a possible pseudogene in a number of laboratory isolates [11], PfRH4 [12] and PfRH5 [3],[12]. EBL homologues include the Duffy Binding Protein (DBP) of *Plasmodium vivax* and *P. knowlesi* [13], the *P. falciparum* EBA175, BAEBL (EBA140), EBL1, JESEBL (EBA181) and PEBL [10], [14]–[18]. These proteins, which are characterized by the presence of the cysteine rich Duffy Binding Like (DBL) domain [13] interact with a wide range of different erythrocyte receptors including Glycophorin A, B and C as well as the Duffy blood group antigen [13], [18]–[22]. However, among the RBL proteins, the erythrocyte-binding properties were only demonstrated for PfRH1, PfRH2b and most recently PfRH4, with the precise binding regions only now being delineated [8],[10],[23].

Erythrocyte invasion by *P. vivax* requires the interaction between the DBP [13] and the erythrocyte Duffy antigen [24] as well as the interaction between the RBP1&2 and an unknown receptor at the reticulocyte surface [25]. The interaction and recognition properties of both EBLs and RBLs seem to define the host cell recognition properties of a merozoite. Numerous studies have indicated that malarial merozoites, especially from *P. falciparum*, can invade erythrocytes through several invasion pathways. This ability is dependent on the repertoire of parasite ligands expressed by the merozoite and variations of receptors at the erythrocyte surface. The various alternative invasion pathways are classified according to the nature of the erythrocyte receptors involved in invasion, which in turn are operationally defined by the enzymatic treatment of erythrocytes that disrupt the specific interactions these ligands make. PfEBA175 is the best characterized receptor and recognizes sialic acid components on Glycophorin A [26], while Glycophorin B, C/D act as receptors for EBLs [18],[27],[28]. Notwithstanding our current limited understanding of the role of RBLs in merozoite invasion, these proteins play an important part in determining host receptor utilization [8],[29]. PfRH1 binds to a neuraminidase-sensitive, chymotrypsin- and trypsin-resistant receptor which has been referred to previously as receptor “Y” [8],[30] whilst PfRH2b recognizes the neuraminidase and trypsin resistant but chymotrypsin sensitive receptor “Z” [31]. PfRH4 binds to a so far uncharacterized neuraminidase and chymotrypsin resistant receptor [32]. Collectively, recognition of a specific receptor at the erythrocyte surface is a crucial step for at least some, if not all RBLs, although direct binding to erythrocytes has been demonstrated only in the case of PvRBP1/2, one member of PY235 and PfRH1 and PfRH4 [8],[10],[23],[25],[33].

How binding of EBLs or RBLs to specific erythrocyte receptors ultimately leads to merozoite invasion is an important question that requires the parasite ligand to be dissected into functional domains. Such an approach has greatly enhanced our understanding of EBL, where the cysteine rich DBL domain was shown to mediate erythrocyte binding [13], while the cytoplasmic domain is required for efficient invasion [34]. Partly because of much lower sequence conservation between members of the RH family, no functional domain such as the erythrocyte-binding region has been well characterized. Recently, however, a 30 kDa recombinant protein from PfRH4 was found to bind erythrocyte but antibodies raised against this region fail to block invasion [23].

Here, we provide the identification of the erythrocyte binding region of the >2700 amino-acid long PfRH1 protein, a member of the RBL family. The recognition domain encompasses only 334 residues predicted to include a N-terminal binding domain and a C-terminal coiled coil region, It binds to erythrocytes in a sialic acid dependent and chymotrypsin/trypsin resistant manner, providing evidence that the main binding determinant is composed of sialic acid residues present within erythrocyte cell surface glycoproteins or glycolipids. Our findings show that only a small segment of the protein is involved in receptor recognition. Thus, it is likely that RBLs mediate other-so far uncharacterized-functions during the invasion process. Epigenetic silencing of *P. falciparum* genes partly regulates the expression of invasion-related ligands and plays an important role in immune response evasion [35]. An antiserum raised against the minimal binding region of PfRH1 contains invasion inhibitory antibodies, and only parasites that utilize a sialic acid-dependent invasion pathway are inhibited by this antiserum. In addition switching of the invasion pathway from a sialic acid-dependent to a sialic acid-independent pathway renders the inhibitory antibodies ineffective with a concomitant reduction in the amount of PfRH1 expressed. Thus, invasion pathway switching in *P. falciparum* can also serve as a mechanism of immune evasion.

## Results

### Defining the erythrocyte binding region of PfRH1 Protein

**Figure 1:**
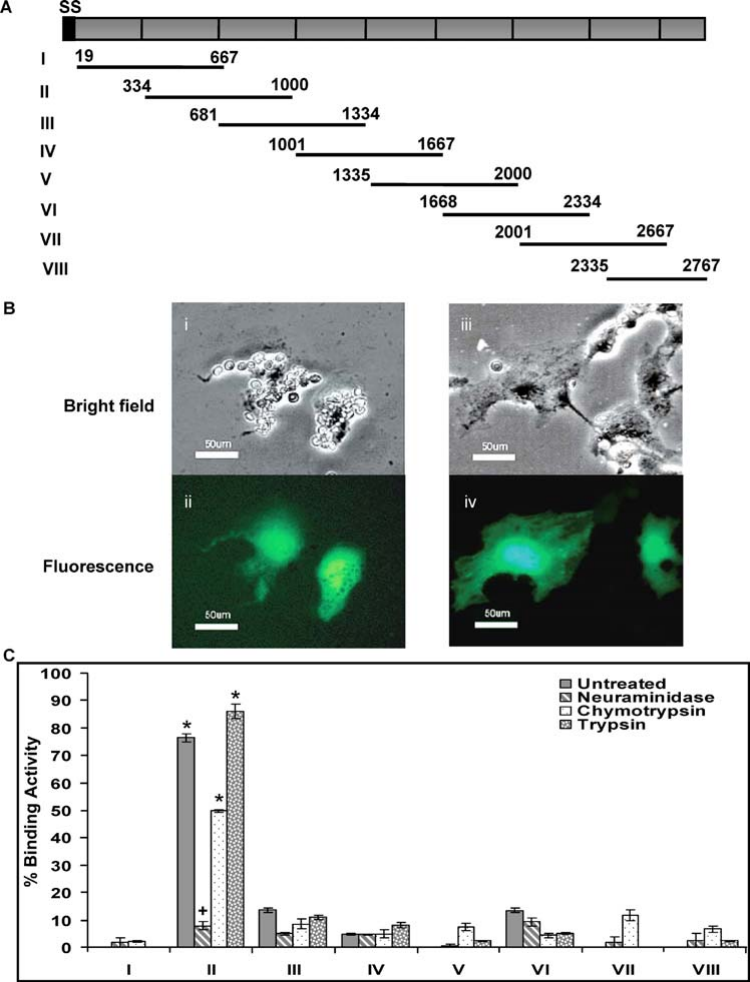
Erythrocyte-binding assay on transfected COS7 cells expressing different regions of PfRH1 before and after enzymatic treatment. (A) Chimeric constructs for the expression of different regions of *PfRH1* in 3D7. 3D7 clone *RH1* DNA sequence (Genbank accession no: AF533700) with intro spliced out includes signal sequence (SS, black) and Exon2 (grey) encoding a large extracellular domain and excludes transmembrane domain and cytoplastic tail. The extracellular domain was divided into eight regions (From I to VIII). Each region (black line with amino acids no.) is approximately 2 kb with 1 kb overlap between 2 consecutive regions except *VIII*, which is 1.3 kb. (B) Duffy-positive human erythrocytes bound to the surface of COS7 cells were visualized as rosettes at a magnification of ×200. (i) Two cells formed rosettes with Duffy-positive human erythrocytes on bright field. (ii) Cell-associated fluorescence as GFP (green) was found on the same field as in panel (i). (iii) Negative count of rosette on transfected COS7 cells on bright field. (iv) But two transfected cells (green) were shown on the same field as in panel (iii). Bar, 50um. (C) Comparison of Erythrocyte-binding assay with different regions of PfRH1 after enzymatic treatment. Rosettes were scored in 30 fields at 200× magnification. Data are present as percentage of binding activity (%) after the number of observed rosettes was normalized to 5% transfection efficiency in three independent experiments. The error bar denotes the SE. * *p*<0.001, indicating that the binding ability of RII is significantly different from those obtained from other regions. + *p*<0.001, indicating that after neuraminidase-treatment, the binding of RII was significantly decreased compared to untreated erythrocytes.

2 kb overlapping fragments of *PfRH1* were cloned into the pRE4 vector (Figure 1A), which has previously been successfully used for expression of a number of different malaria parasite proteins at the surface of COS7 cells [13],[26]. In this vector, the secretory signal sequence and transmembrane segment of HSVgD were used to target different regions of the malarial proteins to the COS7 cell surface [13],[36]. Subsequently, these ∼ 2Kb constructs, together with signal sequence and transmembrane segment, were respectively subcloned into the mammalian cell expression vector pEGFP-N1, to generate GFP fusion proteins [37]. To establish whether the cloned regions had erythrocyte binding activity, COS7 cells were transfected with the GFP constructs. Correct expression of the GFP fusion proteins was monitored by fluorescent microscopy of the transfected cells. COS7 cells transfected with constructs that expressed the different regions of PfRH1 as GFP-fusion proteins, were tested for their ability to bind to human erythrocytes. For each construct, the transfection efficiency was determined by the expression of GFP using fluorescence microscopy (Figure 1B ii and iv). The number of COS7 cells with erythrocytes rosettes was determined in 30 fields, at a magnification of ×200, in three separate experiments. As shown in Figure 1C, region II (amino acids 334–1000) of PfRH1 possesses the strongest binding ability to untreated human erythrocytes with >70% binding activity. Regions III, IV and VI show minimal binding (<10%). No binding was observed for region I, V, VII and VIII of PfRH1. Negative controls with either untransfected COS7 cells, with human erythrocytes or COS7 cells expressing PvDBPII with chymotrypsin-treated human erythrocytes, gave no rosettes (data not shown). To further examine the binding specificity of erythrocytes to region II, we tested the binding ability of all eight constructs described above to neuraminidase-, chymotrypsin- and trypsin-treated human erythrocytes respectively. Previously, it was shown that PfRH1 protein interacts with a neuraminidase-sensitive and trypsin-resistant receptor at the erythrocyte surface [8],[30],[38] and that the binding of EBA-175 is dependent on neuraminidase- and trypsin-sensitive glycophorin A [26]. From three independent experiments, it is clear that region II binding is dramatically affected when erythrocytes are pretreated with neuraminidase (Figure 1C) with binding being reduced approximately 10 fold. Limited or no impact on binding of erythrocytes to region II is seen when the erythrocytes are pretreated with chymotrypsin or trypsin (Figure 1C). Enzyme treatment of erythrocytes had little effect on the minimal binding seen to any of the other regions (Figure 1C). To ensure that the enzyme treatment of erythrocytes was effective, binding of pretreated erythrocytes to COS7 cells expressing either PvDBPII or EBA-175RII [37],[39] was tested. Binding of erythrocytes to PvDBPII is resistant to neuraminidase and trypsin treatment, but sensitive to chymotrypsin. EBA-175RII is neuraminidase and trypsin sensitive but chymotrypsin resistant. For both, the observed binding was as expected, indicating that enzyme treatment was effective (data not shown). Taken together, these data show that region RII of PfRH1 has the expected erythrocyte binding specificity previously demonstrated using the full length protein [8].

**Figure 2:**
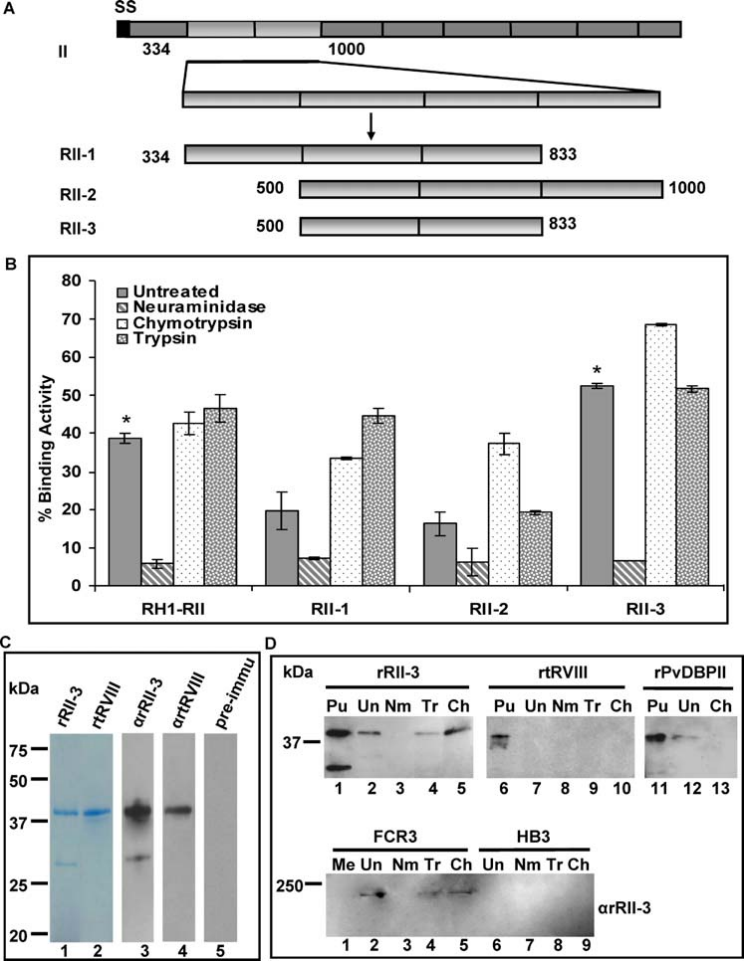
Erythrocyte-binding assay on transfected COS7 cells expressing region II deletion constructs and purified recombinant proteins rRII-3 and rtRVIII. (A) Overall architecture of Region II deletion constructs of PfRH1. RII-1 contains region II amino acids from 334 to 833. RII-2 consists of region II amino acids from 500 to 1000. RII-3 contains region II amino acids from 500 to 833 (light gray). (B) Comparison of erythrocyte-binding assay with full-length RII and deletion constructs before and after enzymatic treatment. Rosettes were scored in 30 fields at 200× magnification. Data are present as percentage of binding activity (%) after the number of observed rosettes was normalized for 5% transfection efficiency in three independent experiments, and the error bar denotes the SE. * *p*<0.001, indicating that after neuraminidase-treatment, the binding of RII and RII-3 was significantly decreased compared to untreated erythrocytes. (C) Purified rRII-3 and rtRVIII were shown in coomassie blue staining (lane 1 and 2) and detected by western blot with αrRII-3, αrtRVIII and pre-immune serum respectively (lane 3 to 5). (D) Purified rRII-3, rtRVIII as well as rPvDBPII bound to erythrocytes was detected by Western blot with anti-His antibody (top). The experiments were carried out from a single batch of purified proteins and run on the same blot. Purified (Pu) rRII-3, rtRVIII and rPvDBPII (Lane 1, 6 and 11). Untreated human erythrocytes (Un) (Lane 2, 7 and 12). Neuraminidase-treated human erythrocytes (Nm) (Lane 3 and 8). Trypsin-treated human erythrocytes (Tr) (Lane 4 and 9).Chymotrypsin-treated human erythrocytes (Ch) (Lane 5, 10 and 13). Enriched culture supernatant from FCR3 and HB3 bound to erythrocytes was also detected by western blot with αrRII-3 (bottom). Culture medium alone (Me) was used as a negative control. The rest of the labels from bottom panel are the same as metioned above. Purified rPvDBPII and culture supernatant were used as positive control to determine the specific binding of the rRII-3. Molecular sizes are indicated on the left (in kDa).

To further delineate the binding region, the same approach for expressing GFP fusion proteins was used to generate deletion constructs RII-1, RII-2 and RII-3 of region II (Figure 2A) [37]. Their expression on COS7 cells was detected by observing the GFP protein expression in fluorescence microscopy. Their binding abilities to human erythrocytes and enzymes-treated human erythrocytes were tested in erythrocyte-binding assays (Figure 2B). Binding was observed with the full-length region II, as well as the deletion constructs RII-1, RII-2 and RII-3 to untreated human erythrocytes. Upon neuraminidase treatment, all the binding activities to human erythrocytes were significantly decreased for RII and the three deletion constructs, whereas treatment with chymotrypsin or trypsin had no inhibitory effect on rosette formation (Figure 2B). For both binding regions of PvDBPII and EBA-175RII, the observed binding was as expected, indicating that enzyme treatment was effective (data not shown). RII-3 showed similar binding to RII, whereas RII-2 and RII-1 had a reduced binding activity, compared to the full length RII protein. The reduction in binding of RII-2 and RII-1 compared to the RII-3 possibly reflects poor folding of the expressed proteins or slightly lower expression levels. Previous studies showed that a single amino acid change can indeed have a significant impact on how the protein is presented at the cell surface [40],[41]. The slight augmentation in binding provoked by chymotrypsin and trypsin treatment is probably due to an increased accessibility of the receptor recognized by PfRH1. Overall, our data suggest that RII-3 contains the minimal binding region of PfRH1.

### Bacterially-expressed recombinant RII-3 specifically binds to erythrocytes

To confirm the finding that RII-3 contains the minimal binding region of PfRH1, the corresponding sequence was cloned into a pET24a(+) vector, containing a 6-His tag at its C terminus and expressed as a soluble protein in *E. coli* (Figure 2C). As a negative control, another pET24a(+) construct expressing a protein of similar size from region VIII (truncated RVIII, encompassing amino acids 2434 to 2767) was prepared. After metal affinity and ion exchange chromatography, the recombinant proteins -named rRII-3 and rtRVIII- were apparent in coomassie-blue staining, with the expected molecular mass of ∼40 kDa (Figure 2C, lane 1 and 2). Antisera against the two recombinant proteins (αrRII-3 and αrtRVIII) were raised in mice and their reactivity with the recombinant proteins was confirmed by Western Blot (Figure 2C, lane 3 and 4). These purified recombinant proteins were subsequently used in erythrocyte binding assays. A recombinant *P. vivax* Duffy Binding Protein DBL domain (rPvDBPII) serving as a positive control [42], binding to untreated or enzyme-treated erythrocytes was performed as described [8],[22],[43]. Bound proteins were run on the same blot and detected by Western blot using an anti-His antibody. A 40 kDa band was found in rRII-3 binding to untreated, chymotrypsin- and trypsin-treated erythrocytes, but not in neuraminidase-treated erythrocytes (Figure 2D, top, lane 2 to lane 5). No binding was detected for rtRVIII either in untreated or enzymes-treated erythrocytes (Figure 2D, top, lane 7 to lane 10). Binding of the rPvDBPII was as expected with the protein binding to untreated but not to chymotrypsin-treated erythrocytes (Figure 2D, top, lane 12 to 13). In order to confirm whether the recombinant protein bound to erythrocytes in the same way as the native PfRH1 protein, supernatants from a parasite either expressing PfRH1 (FCR3) or not (HB3) were used in erythrocyte binding assays (Figure 2D, bottom). PfRH1 binds erythrocytes via a neuraminidase-sensitive and trypsin-, chymotrypsin-resistant receptor [8],[30]. Since identical properties were observed for the PfRH1 molecule detected in the FCR3 culture supernatant, this confirms that the recombinant protein rRII-3 displays the same functional properties as the native PfRH1 protein using the same assays. These results are consistent with the presence of the minimal erythrocyte binding domain of PfRH1 within the RII-3 region.

### A new erythrocyte binding domain in *Plasmodium*

**Figure 3:**
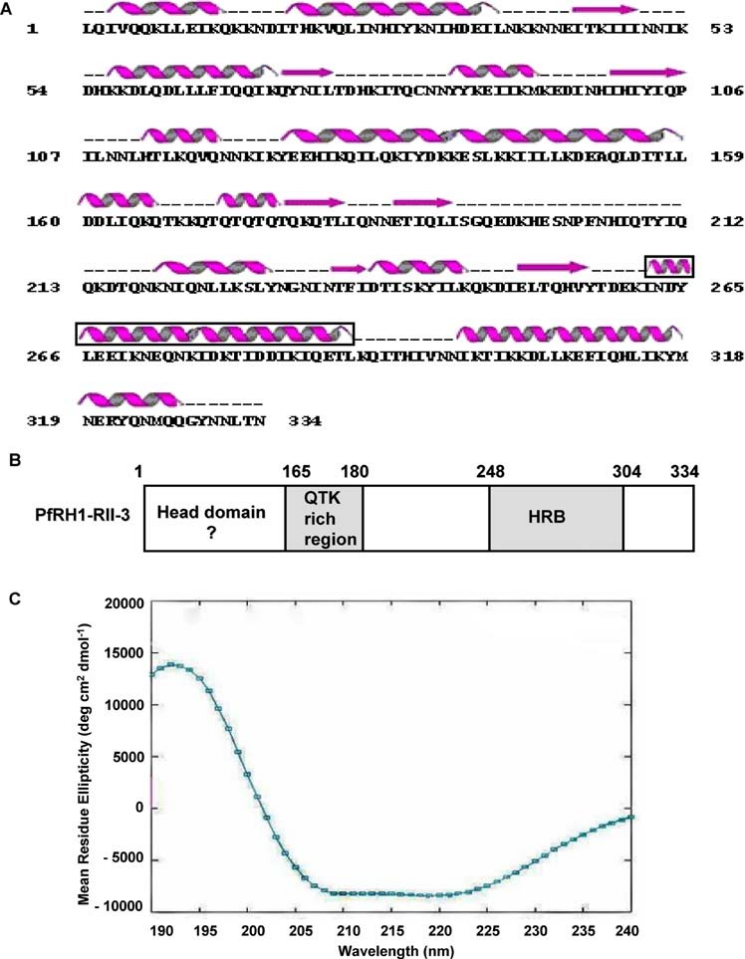
Bioinformatics and biophysical analysis of the minimal binding region RII-3 of PfRH1. (A) Secondary structure prediction of the putative erythrocyte binding region of PfRH1. The predicted secondary structure is shown above the sequence with α-helices as springs and β-strands shown as arrows. The region predicted to form a coiled-coil is boxed and includes residues 262 to 289. Dashed lines indicate regions with no secondary structure prediction. (B) Proposed topology for the RII-3 binding region. HRB indicates the Heptad repeat region which presents weak sequence identity with the parainfluenza F protein heptad repeat B that forms a coiled coil of three-helices. (C) CD spectrum of purified recombinant rRII-3. The graphical output was from SELCON3 (Dichroweb server). The mean residue ellipticity is plotted on the Y axis in degrees cm^2^ dmol^−1^. A positive peak at 190 nm and a negative peak at 208 and 222 nm indicate the presence α-helical structures in the protein.

The RII-3 protein (334 amino-acids) is predicted to be predominantly α-helical (Figure 3A). Circular Dichroic (CD) spectroscopy of the recombinant RII-3 protein was used to assess whether it is properly folded and its secondary structure content. The CD spectrum shows minima at 208 and 222 nm and a positive peak at 190 nm which are characteristic of proteins rich in α-helices (Figure 3C). Spectrum deconvolution indicates a 30% content of α-helices which is consistent with secondary structure prediction programs. Inspection of the RII-3 sequence (Figure 3A) reveals an uneven distribution of amino-acids with a large excess of Ile (16.2%), Lys (14.7%), Gln (12.3%) and Leu (10.2%) residues. The presence of a heptad repeat motif with an Ile side chain at position “a” could be detected between residues 262 to 289 of RII-3 and also in other RB proteins (Figure S1). Furthermore, a weak sequence identity of 27% for 105 aligned amino-acids with the second Heptad repeat region B “HRB” of the parainfluenza virus F protein can be detected between amino-acids 201 to 305 of RII-3 (data not shown). Interestingly the HRB region of the parainfluenza F protein, participates in the formation of a trimeric coiled coil of α-helices [44]. As a confirmation, the program COILS [45] could detect a coiled coil region in the C-terminal domain of RII-3, centered at residue 275 and also in PvRBP-1, PfFRH4, PfRH2a and PfRH3 (Figure S2). To confirm that the RII-3 forms multimers, we performed gel filtration studies using the purified recombinant protein rRII-3. The elution profile from the size-exclusion chromatographic column is consistent with the presence of multimeric as well as monomeric forms of the protein (Figure S3). Further work is needed to confirm the role of the C-terminal coiled coil region in the multimerization of RII-3 and its influence on the binding avidity.

In the absence of a 3D structure, the exact length of the α-helical coiled coil is difficult to assess but is likely to span between 28 to 49 residues which translates into a helix of a length comprised between 43 to 75 Å. By analogy with the parainfluenza F protein, it is thus tempting to describe the RII-3 domain as being composed of an N-terminal “head domain” possibly involved in binding the sialic acid moiety on the erythrocyte surface and a C-terminal multimerization domain mediated by the coiled coil C-terminal region (Figure 3B).

Previous work on the PfRH4 binding region has shown that a recombinant protein containing the binding region can block erythrocyte invasion in a dose dependent manner [23]. To explore whether the rRII-3 recombinant protein had a similar effect we incubated erythrocytes with increasing concentration of protein before the addition of parasites. The rRII-3 inhibited the invasion in a concentration-dependent manner with IC_50_ of 0.39 µM, in contrast no inhibition was observed for rtRVIII (Figure S6A).

### Antibodies raised against recombinant proteins rRII-3 and rtRVIII specifically recognize full length PfRH1

**Figure 4:**
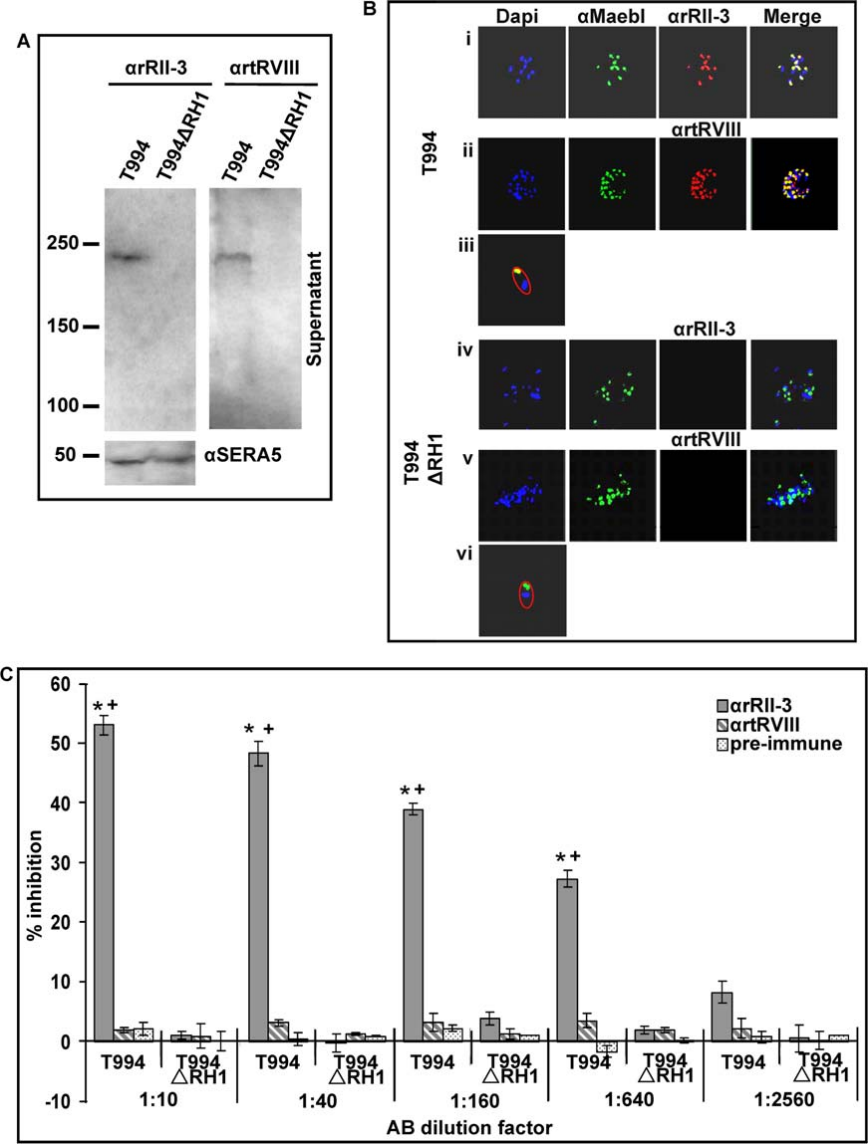
The specificity of mouse polyclonal antisera αrRII-3 and αrtRVIII Immunoblots and single-staining IFA of W2mef with. (A) Western analysis of PfRH1 expression on enriched culture supernatant from T994 and T994ΔRH1 probed with αrRII-3, αrtRVIII and αSERA5 as loading control. The expected protein of about 240 kDa was only detected by both antisera in T994 parasites. Molecular sizes are indicated on the left (in kDa). (B) Double-staining IFAs of T994 and T994ΔRH1 parasites. T994 (panel i, ii and iii) and T994ΔRH1 (panel iv, v and vi). Smear of late stage schizonts and free merozoites were reacted with rabbit polyclonal antiserum αMaebl (second column); αrRII-3 or αrtRVIII (third column). Parasite nuclei are stained with DAPI (blue). For each panel, Alex Flour 488 goat anti-rabbit IgG (H+L) (green) are to rabbit serum, and Alex Flour 594 goat anti-mouse IgG (H+L) (red) to mouse sera. In the merged images, areas of overlap between the red and the green signals are shown in yellow. The outline of single merozoite (panel iii and vi) is shown separately. (C) Comparison of merozoite invasion inhibition with αrRII-3 and αrtRVIII, including pre-immune serum in 1∶10 to 1∶2560 dilutions in T994 and T994ΔRH1. The error bar denotes the SE. * *p*<0.001, indicating that αrRII-3 successfully blocked the invasion in T994 parasites, compared to the invasion in T994ΔRH1 with different dilutions. + *p*<0.001, indicating that αrRII-3 significantly inhibited the invasion compared with αrtRVIII and pre-immune serum.

As earlier studies have given conflicting results about the size of PFRH1 in parasite supernatant (Compare [8] to [30]), we proceeded to further confirm the specificity of the antibodies using culture supernatant obtained from a *P. falciparum* PfRH1 knock-out parasite line T994ΔRH1 as well as its parent line T994 [30] using Western blot analysis. Previous work had shown that an approximately 240 kDa protein is recognized by PfRH1 specific sera in T994 but is completely absent from the knock-out line [30] and an identical result is observed with both antibodies αrRII-3 and αrtRVIII raised here (Figure 4A) with an αSERA5 antiserum serving as a loading control (Figure 4A) [46]. Western blot on schizont and merozoite pellets from T994 and T994ΔRH1 parasites using αg12 antiserum as a loading control [47] confirmed the lack of expression of PfRH1 protein in the knockout parasites and further confirmed the specificity of the two antibodies (Figure S4). Unlike in the parasite supernatant where only a 240 kDa band is detected, αrRII-3 and αrtRVIII detect one additional smaller band each representing processing or degradation products of a larger PfRH1 precursor (Figure S4). This is in line with previous studies [30] showing that the size of PfRH1 detected is lower then the size predicted from the amino acid sequence. Immunofluorescence Assays (IFAs) using a MAEBL specific antibody [48] as a marker for the rhoptries showed that in T994 both αrRII-3 and αrtRVIII gave a punctuate pattern which is clearly localized with MAEBL in the apical end of merozoites consistent with the expected expression pattern of PfRH1 (Figure 4B). No staining was observed in T994ΔRH1 parasites with either αrRII-3 or αrtRVIII (Figure 4B) further confirming the specificity of the two antibodies.

Expression of PfRH1 is linked with the utilization of a sialic acid containing erythrocyte receptor by the parasite during invasion. In line with this invasion of erythrocytes by T994 is inhibited by neuraminidase treatment of erythrocytes, whilst at the same concentration, no inhibitory effect is seen on T994ΔRH1 (Figure S5). The enhanced ability of T994ΔRH1 parasites to invade neuraminidase-treated erythrocytes reflects a shift in these parasites towards the utilization of non sialic acid containing receptors compared with the T994 parent. The different sensitivity of these two parasite clones towards neuraminidase is also reflected in the ability of the RH1 antibodies to inhibit merozoite invasion (Figure 4C). Invasion inhibition assays performed on synchronized cultures showed significant differences in the ability of the αrRII-3 and αrtRVIII antibodies to inhibit invasion. While both the pre-immune serum as well as αrtRVIII had no effect on invasion, at 1∶10 dilution, the antiserum αrRII-3, raised against the minimal binding region of PfRH1, successfully blocked invasion in T994 parent with 53% inhibition, compared to the positive control grown in complete RPMI 1640. The inhibition was concentration-dependent. By contrast, there was very little impact on invasion seen at the same amount of αrRII-3 in T994ΔRH1 parasites. This demonstrates that T994 RH1 knock out parasites are no longer sensitive to the antibodies against the binding region.

The inhibitory effect of the αrRII-3 antibody could be reversed by pre-incubation of the antiserum with the recombinant rRII-3 protein. Addition of as little as 3.12 µm of rRII-3 reduced invasion inhibition from approximately 55% to 40% while the addition of 25 µm of protein reduced invasion inhibition to around 10% (Figure S6B). In contrast pre-incubation with rtRVIII had no effect on invasion with invasion inhibition still being >50% even after the addition of 25 µm of protein (Figure S6B). The ability of rRII-3 to reverse the invasion inhibitory effect of the αrRII-3 antiserum in a dose dependent fashion further confirms the specificity of this antibody.

### Inhibition of invasion by *P. falciparum* parasites with antisera targeting different regions of PfRH1

**Figure 5:**
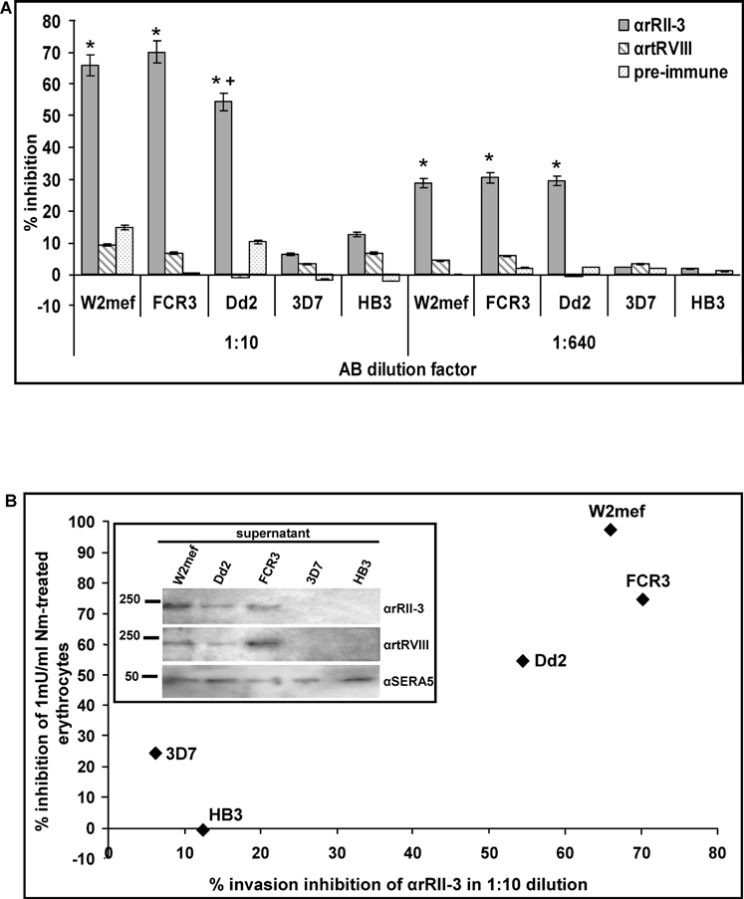
Merozoite invasion properties of *P. falciparum* W2mef, FCR3, Dd2, 3D7 and HB3 clones. (A) Comparison of merozoite invasion inhibition with αrRII-3 and αrtRVIII, including pre-immune serum in 1∶10 and 1∶640 dilutions. Positive control was the invasion of merozoites into the normal complete medium. The error bar denotes the SE. * *p*<0.001, indicating that the invasion of W2mef, FCR3 and Dd2 were successfully inhibited by αrRII-3, compared to 3D7 and HB3. + *p*<0.05 indicating that Dd2 exhibit an intermediate sensitivity to inhibitory antiserum as compared to W2mef and FCR3. (B) Parasites were also assayed for their ability to invade neuraminidase-treated erythrocytes (1mU/ml final). For each parasite clone, the percentage of inhibition of the ability to invade neuraminidase-treated erythrocytes (vertical axis) is plotted against the percentage of invasion inhibition of αrRII-3 in a 1∶10 dilution (horizonal axis). Western blot of enriched culture supernatant from all 5 clones used in this study and probed with αrRII-3, αrtRVIII and αSERA5 was shown in the insert of (B). No RH1 protein was detected in 3D7 and HB3 clones compared with W2mef, Dd2 and FCR3.

Previous work demonstrated that different *P. falciparum* parasite clones invade either via a sialic acid-dependent or independent pathway and the redundancy in the RBL family of proteins at the level of gene number and sequence and the variations in transcription and protein expression may allow the parasite to use alternative invasion pathways [49]. PfRH1 plays an important role in sialic acid-dependent invasion [30]. We therefore investigated whether the various anti-RH1 sera exhibited different effects on invasion by different parasites clones (Figure 5A). As seen with the T994 parasite clone, neither the pre-immune serum nor αrtRVIII had a significant effect in an invasion inhibition assays performed on synchronized cultures of five clones. By contrast αrRII-3 showed dramatic differences in its ability to inhibit invasion. At a 1∶10 dilution, αrRII-3 blocked invasion in W2mef, FCR3 and Dd2 at levels of 66%, 70% and 54% respectively and at a 1∶640 dilution, approximately 30% inhibition was observed for all 3 clones, compared to the positive control. By contrast there was very little impact on invasion seen at the same amount of αrRII-3 in 3D7 and HB3 clones. The invasion inhibition assay demonstrates that antiserum raised against the binding domain of PfRH1 (RII-3) contains more efficient invasion inhibitory antibodies then those raised against another region of the protein.

To establish whether there was any link between antibody sensitivity and sialic acid dependent invasion we determined the sensitivity of the different parasite clones to neuraminidase-treated erythrocytes. 3D7 and HB3 were able to invade neuraminidase-treated erythrocytes more efficiently than W2mef, FCR3 and Dd2 (Figure 5B). Neuraminidase inhibited the invasion of W2mef, FCR3 and Dd2 at low concentrations (0.01-1 mU/ml), but had minimal to no effect on 3D7 and HB3 clones (Figure 5B and data not shown). Treatment of erythrocytes with 1 mU/ml neuraminidase inhibited invasion of W2mef, FCR3 and Dd2 at levels ranging from 56% to 97%, whereas the inhibition for 3D7, HB3 was 0 to 24%. The parasite clone Dd2 exhibits an intermediate sensitivity to neuraminidase treatment compared to W2mef and FCR3. This is reflected in the slightly lower sensitivity to the invasion inhibitory antibodies (Figure 5B). To determine whether the differential sensitivity to αrRII-3 antibodies reflected variations in PfRH1 expression in the different *P. falciparum* clones, we performed quantitative Western blots on both culture supernatants and merozoite extracts using αrRII-3 and αrtRVIII (Figure 5B insert and Figure S7). A protein band of about 240 kDa was readily detected with both antibodies in supernatant from W2mef, Dd2 and FCR3, but not from 3D7 and HB3, when compared with the control protein SERA5 (Figure 5B insert). Dd2 seems to express an intermediate RH1 level as compared to W2mef and FCR3. To confirm this observation, a Western blot of merozoite extract from the same clones was also probed with αrRII-3 and αrtRVIII (Figure S7). Identical results were obtained using merozoites from W2mef, Dd2 and FCR3, except that a very small amount of protein could be detected in 3D7 (Figure S7). Taken together, our data show that parasites that use a sialic acid-dependent invasion pathway are sensitive to antibodies targeting the PfRH1 binding domain.

### Changes in PfRH1 in W2mef and W2mef (switched) parasites

**Figure 6:**
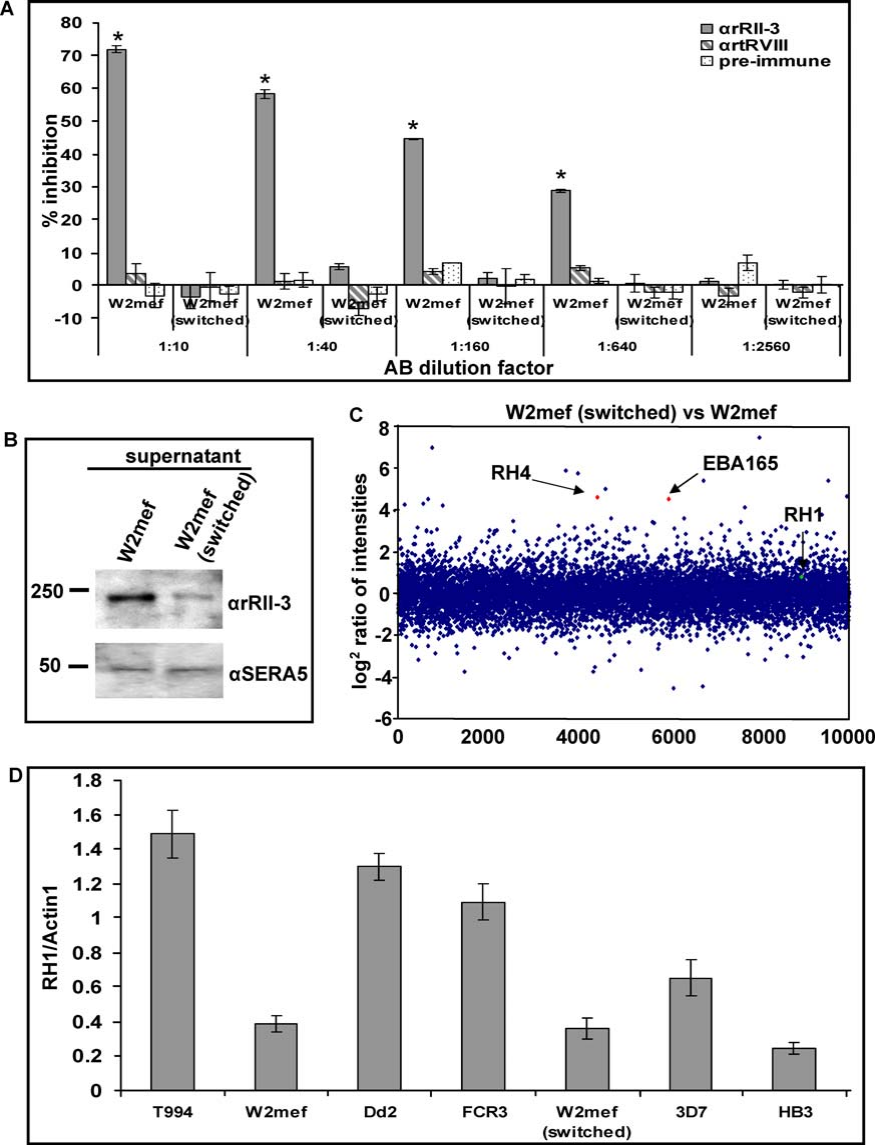
Comparison of PfRH1 expression in merozoite of W2mef and W2mef (switched). (A) Invasion inhibition assay of W2mef and W2mef (switched) with αrRII-3 and αrtRVIII, including pre-immune serum in 1∶10 to 1∶2560 dilutions. The invasion of W2mef merozoites was dramatically inhibited by αrRII-3. The error bar denotes the SE. * *p*<0.001, indicating that the invasion of W2mef parasite was dramatically inhibited by the αrRII-3 under different dilutions, compared with W2mef (switched) parasites. (B) Enriched culture supernatant from W2mef and W2mef (switched) parasite were probed with αrRII-3 and αSERA5. There was higher decreased in RH1 expression level in W2mef (switched) parasites. Molecular sizes are indicated on the left (in kDa). (C) Microarray comparison of gene expression intensity between RNA extracted from synchronized late stage parasites (late trophozoites to schizonts stages) of W2mef and W2mef (switched) parasite. Upregulation of RH4 and EBA165 are shown as red spots. RH1, shown as green spot, in the W2mef (switched) is similar to the transcription in W2mef. The scatter plot represents log_2_ expression ratios between the swiched parasite line (positive values) and its parental line (negative values) (y axis) for all oligonucleotide elements (random order along×axis) for which the hybridization intensity signal passed the filtering criteria (see material and methods) (D) Real-time PCR. Gene expression in parasite lines used in this study is expressed as comparing the Ct (cycle threshold) value for each gene of interest (*PfRH1*) with that of the housekeeping gene (*Actin1*) based on the specific standard curve. The results are from three independent experiments, and the error bar denotes the SE.

The parasite clone W2mef has the capacity to switch from sialic acid-dependent to –independent invasion by selection on neuraminidase-treated erythrocytes [29],[50],[51]. For instance, W2mef Δ175 parasites can switch from sialic acid-dependent to –independent invasion [29],[51]. To obtain W2mef (switched) parasites able to invade erythrocytes via a sialic acid independent pathway we cultured W2mef parasites in the neuraminidase-treated erythrocytes as previously described [32]. The ability of the αrRII-3 and αrtRVIII antibodies to inhibit invasion of W2mef and W2mef (switched) parasites was determined (Figure 6A). Unlike W2mef where invasion was inhibited 71% at a 1∶10 dilution and around 30% at a 1∶640 dilution of αrRII-3 no effect was found in W2mef (switched). As expected, the W2mef (switched) but not W2mef parasites were able to invade neuraminidase-treated erythrocytes efficiently (Figure S8). These data confirm that the parasites utilizing a sialic acid-dependent invasion pathway are sensitive to the antibody targeting the PfRH1 binding domain.

As PfRH1 expression levels are low in parasites less sensitive to the αrRII-3 sera, we investigated its expression level using Western Blot analysis of equivalent amounts of culture supernatants from either parasite population. αrRII-3 recognizes the appropriate size (240 kDa) protein in both parasites (Figure 6B). The level of PfRH1 expression is significantly reduced in switched W2mef as compared to W2mef. Likewise, a lower amount of expressed PfRH1 proteins is seen in schizont and merozoite extracts of switched W2mef parasites (Figure S9). PfRH4 is up-regulated when parasites switch to a sialic acid-independent invasion pathway [32],[52] while no apparent change in the transcript levels of PfRH1 was observed. To confirm that this was also the case in our parasite samples, we performed microarray analysis of RNA extracted from synchronized schizonts. As previously reported, both PfRH4 and EBA165 are significantly up-regulated in the W2mef (switched) parasites but no change was found in the levels of PfRH1 transcription (Figure 6C). This was confirmed using quantitative real-time polymerase chain reaction (RT-PCR) (Figure 6D). To investigate whether other parasites showed a similar independence of PfRH1 transcription and expression, quantitative RT-PCR was performed on all the other parasite clones used in this study (Figure 6D). Parasite clones T994, Dd2 and FCR3 have relatively high PfRH1 levels of transcription, 3D7 has an intermediate transcriptional level and W2mef, W2mef (switched) and HB3 have low transcriptional levels. The maximum difference between the lowest and highest transcription levels of PfRH1 is approximately 7-fold (compare Figure 6D, T994 vs HB3). These data indicate an absence of correlation between RH1 transcription and expression. 3D7 has an intermediate transcriptional level of RH1, but has no expression of RH1. W2mef has low transcriptional level of RH1, but has high expression level of RH1.

## Discussion

The invasion of erythrocytes by *Plasmodium* merozoites is a multi-step process that involves a sequence of molecular interactions. The early steps of erythrocyte invasion include: (a) initial attachment, (b) apical reorientation, and (c) junction formation that initiate entry into the vacuole [53]. Once the merozoite–erythrocyte junction is initiated, the next phase begins with the movement of the junction around the penetrating merozoite. This involves a number of molecular events that allow the merozoite to gain physical entry into the erythrocyte within the parasitophorous vacuole. Parasite ligands encoded by the EBL and RBL gene family mediate interactions that lead to host cell recognition and junction formation. Based on work done in *P. vivax* RBP-1/2, the RBLs have been implicated in the initial recognition of the appropriate host-cell, while DBP has been proposed to be important in the final high affinity binding and tight junction formation. Recent work in *P. knowlesi* has experimentally confirmed the role of the PkDBP in junction formation [54]. At least one member of both RBLs and EBLs is found in all *Plasmodium spp.* analyzed to date, consistent with conserved but distinct functions of both protein families in merozoite invasion.

Significant progress in the study of these ligands was made after the identification of the relatively conserved DBL domain responsible for receptor binding of the EBL superfamily to the erythrocyte. This has enhanced our understanding of the functional role played by this protein during the invasion process. Furthermore, the identification of the DBL domain enabled studies of its role in immune evasion and highlighted the possibility of using this region for the development of a vaccine that would block invasion.

Unfortunately, to date, the high sequence diversity of the RBL superfamily has made it difficult to predict functional regions within these very large proteins. Considering that RBLs range in size from around 200 kDa to more than 350 kDa, the study of the various functions that the RBLs play during merozoite invasion has been significantly hampered. To address this issue, we used several approaches to identify the erythrocyte binding region of one member, PfRH1. Expression of different regions of PfRH1 in both COS7 cells assays as well as recombinant proteins in *E. coli* allowed us to identify an approximately 300 amino-acids erythrocyte-binding domain that retains the binding properties reported for the full length PfRH1 protein [8]. Having defined the binding region of PfRH1 now opens up the prospect of further structural studies to map the binding determinants on the RII-3 protein structure and its interactions with sialic acid residues on the erythrocyte receptor. The PfRH1 binding region and also other members of the PfRBLs family (see Figure S1) contain a coiled coil motif at their C-terminal end possibly involved in protein multimerization and the presence of a trimeric complex in the recombinant protein is consistent with such a prediction. We propose that the N-terminal end of RII-3 forms a “head” domain responsible for binding sialic acid residues at the surface of glycoproteins or glycolipids anchored at the erythrocyte surface. Support for this role played by the N-terminal region comes from the recent identification of a PfRH4 binding region which overlaps by about 89 amino acids with the “head” domain of the PfRH1 binding region [23]. It is now possible to perform detailed studies on the receptor ligand interactions for at least one member of the RBL family, enabling us to address this hypothesis. It is apparent from our study that the region directly involved in erythrocyte receptor binding constitutes only a relatively small region of the full-length protein, like in the case of the EBL protein. Further studies should address the role played by the remainder of the PfRH1 protein. Receptor binding ultimately leads to signaling events that allow invasion and junction formation. Defining the binding region of one member of RBL is an essential first step in understanding this cascade of events.

Work on EBLs has shown that antibodies against the DBL domain are potent inhibitors of invasion. These studies have encouraged significant efforts in developing the DBL domain of PfEBA-175 and PvDBP as part of a malaria vaccine formulation. While previous studies have shown that antibodies against different regions of PfRH1 and PfRH2b [8],[31] can inhibit merozoite invasion of trypsin treated erythrocytes, our work demonstrates for the first time that antibodies against the functionally active PfRH1 binding region contain antibodies that are able to efficiently inhibit invasion of untreated erythrocytes. Considering that both RBL and EBL function sequentially during merozoite invasion, a vaccine targeting both ligands could be more efficient.

The invasion pathways used by *P. falciparum* vary both in laboratory isolates as well as in field isolates [38],[55],[56]. Different parasites display a relatively stable expression profile of RBLs and EBLs that correlates with the invasion pathway used [57]. Here we show that invasion by parasite clones known to use sialic acid-independent receptors is not affected by antibodies raised against the PfRH1 binding region as opposed to parasites using sialic acid dependent receptors. This is consistent with previous observations that parasites using sialic acid-independent invasion often express low levels of PfRH1 or -as in the case of 3D7- a nonfunctional protein [8],[30]. High levels of PfRH1 expression correlates strongly with sialic acid-dependent invasion [30]. Importantly, our findings indicate that the use of alternative invasion pathways is indeed associated with immune evasion as it renders invasion inhibitory antibodies raised against a specific member of RBLs ineffective.

Previous studies of the W2mef parasite clone demonstrated that a switch from sialic acid -dependent to independent invasion is associated with the up-regulation of PfRH4 expression, whilst no changes in PfRH1 transcription was observed [32]. Our microarray and quantitative RT-PCR data are consistent with this observation. Using anti-RH1 antibodies raised against the binding domain, we found that W2mef merozoite invasion is inhibited whereas very little impact on invasion using the W2mef (switched) parasite is seen. Genetic disruption of RBL and EBL molecules leads to an altered invasion pathway by merozoites [32] and has a direct impact on the ability of antibodies to inhibit merozoite invasion [31],[57]. This indicated that invasion pathway switching by the malaria merozoite could provoke immune evasion, although a direct demonstration using non-genetically manipulated parasites was hitherto lacking. Our findings demonstrate for the first time that switching of the invasion pathway by *P. falciparum* clones can constitute an effective mechanism for immune evasion. Previous studies have proposed a “limited space hypothesis” in which the spatial arrangement of parasite ligands at the apical end of the merozoite defines whether a ligand is actively involved in invasion or not [30],[31]. This model as well as the transcriptional data obtained by Stubbs [32] suggest that invasion pathway switching by W2mef from sialic acid-dependent to independent in addition to up-regulation of PfRH4 also leads to reorganization of PfRH1 at the apical end, to a position that makes it inaccessible to blocking antibodies. Our data on the other hand, suggest that switching by W2mef involves a reduction of the overall expression levels of PfRH1, reducing the contribution this protein has on defining the invasion pathway and thereby also reducing the effectiveness of the inhibitory antibody. The fact that there is still some PfRH1 detected by Western blot in merozoites indicates that expression is not completely eliminated. Therefore, the possibility exists that these two mechanisms may well work in tandem.

Up-regulation of PfRH4 is also observed in W2mef in the case of genetic disruption of EBA175 [32], suggesting some functional redundancy between RBLs and EBLs. Our results on the other hand, would indicate that up-regulation of PfRH4 is a direct consequence from the parasite switch to a sialic acid-independent invasion pathway. Whether changes in the expression of different EBLs -as seen here for PfRH1- plays a role in the case of the EBA175 knockout requires further investigations. Further work is also needed to address the influence between both invasion protein families in more detail.

To a large extent, transcription and expression levels for RBLs seem to correlate reasonably well, although this does not seem to be the case for PfRH1 in W2mef (switched) parasites, where PfRH1 transcription levels do not change significantly when the parasite switches invasion pathways [32], despite a significant reduction in the overall expression of PfRH1 protein. In addition the quantitative RT-PCR data presented here indicate that while overall transcriptional levels are somewhat higher in parasite clones expressing significant amounts of PfRH1, this is not always the case (See 3D7, W2mef and W2mef (switched)). Epigenetic and post-transcriptional mechanisms play an important role in expression control in *Plasmodium* [35]. This seems to be true for the regulation of PfRH1, where post-transcriptional mechanisms regulate protein levels and thereby modulate the invasion properties of merozoites. Thus, a better understanding of how transcription and translation of invasion molecules are coupled is essential to reveal the mechanisms that regulate merozoite invasion pathways and merozoite immune evasion. All clones of *P. falciparum* express multiple invasion ligands and there is a functional hierarchy, in which some ligands are dominant over others [57]. The identification of the erythrocyte binding domain of a PfRH is an important step in dissecting the molecular function of these proteins. We show that the binding domain could serve as an effective vaccine candidate against parasite expressing this ligand, although the ability of the parasite to switch invasion pathways and therefore circumvent the effect of these antibodies indicates that additional components are required to make such a vaccine efficient. We further demonstrate that the activation of the sialic acid-independent invasion pathway in *P. falciparum* not only requires differential gene expression involving *PfRH4,* but also post-transcriptional regulation of PfRH1. Our data suggest that the increase in expression of PfRH4 in parasites that have switched to a sialic acid-independent pathway is to compensate for the loss of PfRH1. Importantly, variations in the expression of RBLs are a clear mechanism by which a merozoite can evade host immunity.

## Materials and Methods

### Constructs for Expression of Different Regions of PfRH1 on Surface of COS7 Cells

PCR products (for primers see Protocol S1, S1.1–S1.3) where amplified from *P. falciparum* 3D7 genomic DNA (gDNA) and cloned into pRE4 and pEGFP-N1 vector [37] so as to express the recombinant fusion protein to N- terminus of the EGFP as a transfection marker.

### Erythrocyte-Binding Assay on COS7 Cells

This assay was carried out as described earlier [13]. Transfected COS7 cells with at least half their surface area covered by erythrocytes were scored as positive for binding [58]. The number of rosettes was counted in 30 fields at 200X magnification using an inverted fluorescent microscope [37],[59]. In each experiment, two wells of COS7 cells were transfected for each construct, and the data shown are from at least 2 separate experiments. The transfection efficiency (%) was calculated as total no. of fluorescent COS7 cells×100/total no. of COS7 cells, while binding activity (%) was calculated as total no. of fluorescent COS7 cells with rosettes×100/total no. of COS7 cells. The number of rosettes observed was normalized for transfection efficiency of 5% [43],[60]. Binding was scored as negative when no rosettes were seen in the entire well. Experimental data are presented as the mean±SE. One way analysis of variance (ANOVA) was used with a post hoc (Bonferroni) test to determine the difference between regions. The significance level was set at *p*<0.05.

### Expression and Purification of Recombinant Proteins rRII-3, rtRVIII and rPvDBPII in *E. coli*

DNA sequence of RII-3 and tRVIII recombinant constructs were amplified by PCR using primers as shown in Protocol S1, S1.4. The PCR products were digested with *EcoR*I and *Xho*I and cloned into expression vector pET24a(+) (Novagen) to generate a C-terminal His-tag. BL21-CodonPlus-RIL (Stratagene) was used to express recombinant rRII-3 and rtRVIII. IPTG at a final concentration of 0.2mM was added to cultures at A_600 nm_ of 0.6 –0.8. Induced cultures were allowed to grow overnight at 16°C and then resuspended in chilled lysis buffer (50 mM Tris, pH 8.0, 200 mM NaCl, 0.1% tween-20 with protease inhibitor cocktail, EDTA-free (Roche) for rRII-3; 50 mM Tris, pH 8.5, 200 mM NaCl, with protease inhibitor cocktail, EDTA-free for rtRVIII), and lysed by sonication. The recombinant protein was purified under native conditions using nickel-nitrilotriacetic acid-agarose (Ni-NTA) (Qiagen), followed by ion-exchange chromatography using a MonoS HR 5/5 column (Amersham) for rRII-3 and MonoQ 5/50 GL column (Amersham) for rtRVIII.

DNA encoding DBPII (amino acids from 194 to 521 of *P. vivax* Duffy-binding protein) was amplified by PCR with primers 5′-GCG CGA GCT CTT ATG TCA CAA CTT CCT GAG TAT TTT T-3′ and 5′- GTC GCC ATG GGA GAT CAT AAG AAA ACG ATC TCT AGT-3′ using DBPIIpETDEST42 as template. The PCR product was digested with NcoI and SacI and cloned into modified expression vector pET9d (personal contributed by Prof. Gruber's lab) to generate N-terminal His-tag. The correct product was then transformed into BL21-Rosetta-gami for expression of recombinant DBPII (rPvDBPII). rPvDBPII was induced at same condition mentioned above and purified under native conditions using Ni-NTA, followed by gel filtration chromatography using Superdex 75 Column (Amersham) in PBS, pH7.4, with a 180 mM NaCl.

### Analysis of Secondary Structure Content of Recombinant Proteins rRII-3 by Circular Dichroic (CD) Spectroscopy

The CD spectra were recorded on a Jasco (J-810) spectro-polarimeter. Spectra of purified rRII-3 in 50 mM HEPES, pH 7.1, containing 150 mM NaCl, 50 µm L-arginine, were recorded at concentration of 200 µg/ml by using three accumulations of data at 0.1 nm intervals. Solvent background was subtracted and the data processed by the interactive Dichroweb server [61] using the program SELCON3. The mean residue ellipticity is plotted on the Y axis with units degrees.cm^2^ .dmol^−1^. The root mean square deviation between observed and deconvoluted data is 0.115.

### Western Blot of Parasites Extracts and Culture Supernatants

Tightly synchronized late schizonts (greater than 80% parasitaemia), or purified merozoites were collected as described (http://www.lumc.nl/1040/research/malaria/ malaria.html). The schizonts or merozoite extracts were lysed directly into sample buffer and frozen and thawed 3 times. The supernatants were used for SDS-PAGE using% gels (for PfRH1 protein) and 12% gel (for probed with αg12). To make culture supernatant, purified schizonts were placed back into culture containing only one-fourth of original volume of complete medium. Cells were harvested by centrifugation after 16hrs and supernatant were stored in aliquots at −80C°. The culture supernatant was separated on 6% SDS-PAGE (for RH1 protein) and 10% gel (for SERA5 protein). Following electrophoresis, gels were transferred onto nitrocellulose membranes (0.2um) (Bio-Rad)). Specific proteins were detected by using polyclonal antisera against rRII-3 and rtRVIII, or mouse monoclonal antiserum g12 raised against a 34 kDa protein (homologue of the HSP90 co-chaperone p23) [47] for loading control, or mouse pre-immune serum to check the specificity, followed by horseradish peroxidase-linked secondary antibodies (Sigma) and enhanced chemiluminescence (Pierce).

### IFAs and Microscopy

Synchronized late-stage schizonts were smeared and air-dried followed by fixation with 2.5% Glutaraldehyde (Sigma) for 30min at room temperature. Slides were rinsed in PBS and immersed in blocking buffer (4% bovine serum albumin and 0.5% Triton X-100 in PBS) at 37°C for 30 min in a humid chamber. Smears were incubated with mouse antisera either αrRII-3 or αrtRVIII and co-incubated with rabbit antiserum to PfMaebl [48] for 30 min in blocking buffer, followed by three 5-min washes in 0.05%Tween-20 in PBS. Mouse pre-immune serum was also used in this experiment. They were then incubated with a mixture of Alex Flour 594 goat anti-mouse IgG (H+L) and Alex Flour 488 goat anti-rabbit IgG (H+L) secondary antibodies (Molecular Probe) for 30 min. Slides were washed in 0.05%Tween-20 in PBS for three 5-min, and dipped in diamidinophenylindole (DAPI, 0.5ug/ml), and washed in 0.05%Tween-20 in PBS for four 1-min. Vectorshield mounting medium (Vector laboratories) was applied to the slides, and the coverslips were sealed. The fluorescence images were captured using a LSM510 Confocal Microscopy (Zeiss).

### Invasion Inhibition Assay

Synchronized late stage schizonts were purified [62] and 160ul of parasites suspension was added in duplicated in a 96 well flat-bottom microtitre plate containing 40ul of serial dilution from 1∶10 to 1∶2560 of either αrRII-3, αrtRVIII or pre-immune serum. 1,000 to 2,000 erythrocytes were scored for presence of rings on Giemsa-stained smears after 24 h for reinvasion. Invasion was present as (%) parasitaemia. Parasitaemia (%) = total no. of RBCs infected with rings/total of RBCs×100. Invasion in the presence of antisera was compared with positive control of invasion of the same parasite clones into the normal complete RPMI 1640. Invasion inhibition efficiencies were determined as follows: Inhibition efficiency (%) = (1−Inv(antisera)_dilu_)/Inv(positive))×100. Data shown are from two separate experiments. Experimental data are presented as the mean±SE. One way analysis of variance (ANOVA) was used with a post hoc (Bonferroni) test to determine the different effect of the antisera between parasite lines. The significance level was set at *p*<0.05.

### RNA Purification, Quantitative Real-time PCR and microarray analysis

Schizont stage parasites were separated and harvested with Percoll and total RNA was extracted from schizonts using the Total RNA Mini Kit (Geneaid). For quantitative real time RT-PCR isolated total RNA was digested with DNase I (Fermentas) before converting to cDNA using Invitrogen Superscript First Strand Synthesis System kit (Invitrogen). 1ng of cDNA in 15 µl reaction volume was amplified using Sybr Green Master mix (Applied biosystems) with gene of interest, or housekeeping gene, followed by analyzing on an ABI 7000 thermocycler under the cyling conditions: 50°C for 2min, 95°C for 10 min, 9°C for 1min, 53°C for 1min, 60°C for 1min, 95°C for 15sec and 60°C for 1min. Genes were amplified from genomic DNA extracted from 3D7 and cloned into TOPO-TA cloning vector (Invitrogen) to generate standard curves for quantitative real-time PCR. Water and no-reverse transcriptase controls were included in each real-time PCR and each gene was analyzed in triplicate using RNA from two independent RNA isolations. Transcriptional level was determined by comparing the Ct (cycle threshold) value for each gene of interest with that of the housekeeping gene based on the specific standard curve as previously described [32],[63]. The data are from three independent experiments, and presented as the mean±SE.

Primers used included:

PfRH1: 5′ GATAAAGAGCAAGAAAAACAACAAC 3′ and 5′ CATTACCTCTTCTTG ATTTCTACCA 3′
Actin1: 5′TGCACCACCAGAGAGAAAAT 3′ and 5′ ACTTGGTCCTGATTCATCGT 3′.

For the transcriptional profiling we utilized a long oligonucleotide microarray containing 10166 microarray elements representing 5363 *P. falciparum* genes [64]. The microarray hybridizations were performed using the standard protocol as previously described [65] with small modifications. Briefly RNA was isolated from synchronous W2mef and W2mef (switched) late stage parasites as previously described [32]. For the hybridization W2mef or W2mef (switched) cDNA were coupled to Cy5 fluorophore and hybridized against the 3D7 reference pool (coupled to Cy3). The reference pool consists of RNA samples representing all developmental stages of the parasite. For each hybridisation, 12 µg of pooled reference RNA or sample RNA was used for cDNA reaction. Microarray hybridisations were incubated for 14–16 h using Maui hybridization system (Bio Micro Systems). Data were acquired and analyzed by GenePix Pro 3 (Axon Instruments). Array data were stored and normalised using the NOMAD microarray database (http://ucsf-nomad.sourceforge.net/). The final dataset was filtered as follows: total signal intensity of each spot must be greater than local background plus one standard deviation of the background (Dataset S1).

### Accession Numbers

The PlasmoDB (http://plasmodb.org/PlasmoDB.shtml) accession numbers for the genes studied in this paper are *PfRh1* (PFD0110W).

## Supporting Information

Dataset S1 Log transformed data of the microarray analysis of schizont stage RNA of W2mef vs W2mef (switched)
(1.68 MB XLS)

Protocol S1 Additional material and methods used in this study
(0.07 MB DOC)

Figure S1 Sequence alignment of putative binding regions of different RH members
(0.94 MB DOC)

Figure S2 Secondary Structure Prediction of minimal binding region
(0.42 MB DOC)

Figure S3 Size exclusion chromatography of recombinant RII-3
(0.52 MB DOC)

Figure S4 Western blot of T994 and T994ΔRH1 extracts using anti-RH1 antibodies
(0.38 MB DOC)

Figure S5 Invasion of T994 and T994ΔRH1 into neuraminidasse-treated erythrocytes
(0.32 MB DOC)

Figure S6 Invasion inhibition assay and invasion competition assay with rRII-3 in W2mef
(0.44 MB DOC)

Figure S7 Western blot of merozoite extracts from different parasite lines using anti-RH1 antibodies
(0.28 MB DOC)

Figure S8 Invasion of W2mef and W2mef (switched) parasites into neuraminidasse-treated erythrocytes
(0.40 MB DOC)

Figure S9 Western blot of W2mef and W2mef (switched) merozoite and schizont extracts probed with anti-RH1 antibodies
(0.19 MB DOC)
