# Supplementary material for: Antibodies Targeting the PfRH1 Binding Domain Inhibit Invasion of Plasmodium falciparum Merozoites
Source: PLoS Pathog. 2008 Jul 11;4(7):e1000104. doi: 10.1371/journal.ppat.1000104 (PMC2438614; doi:10.1371/journal.ppat.1000104)
Supplement: Protocol S1 — Additional material and methods used in this study (0.07 MB DOC) [file ppat.1000104.s002.doc]

**Protocol S1**

**S1. PCR Primers Sequences for all Constructs in this paper**

S1.1. PCR primers for different regions of PfRH1 designed for pRE4 vector

I: Contains amino acids from 19 to 667. The primers 5’ G ACCAGCTGgaatttagccatgaacaggaa 3’ and 5’AACGGGCCCTTTTGTTTG CTTTTGTATTAA 3’ were used for PCR amplification.

II: Contains amino acids from 334 to 1000. The primers 5’ GACCAGCTGAAAGATGTAATAAATAATAAG 3’ and 5’ AACGGGCCCATTTAA TATTTTTAAAATTTT 3’ were used for PCR amplification.

III: Contains amino acids from 681 to 1334. The primers 5’ TCTCGTCAGCTGCTAATACAAAATAATGAGACG 3’ and 5’ ACGATGGGGCC CTATATCGTCAAAATGTTTTGT 3’ were used for PCR amplification.

IV: Contains amino acids from 1001 to 1667. The primers 5’ GACCAGCTGATATCCTTAAAAGCTTGTGAA 3’ and 5’ AACGGGCCCTTTAGA TTTGTTTACATCTAT 3’ were used for PCR amplification.

V: Contains amino acids from 1335 to 2000. The primers 5’ GACCAGCTGTACCATGCTGATGATACACGT 3’ and 5’ AACGGGCCCTATAAA AACATTATATATTTC 3’ were used for PCR amplification.

VI: Contains amino acids from 1668 to 2334. The primers 5’ GACCAGCTGAATAATGCTCAACTATATTTT 3’ and 5’ AACGGGCCCATTCAT TTGTTCTAATTTGTT 3’ were used for PCR amplification.

VII: Contains amino acids from 2001 to 2667. The primers 5’ GACCAGCTGCAATCATATAATTTAATACAA 3’ and 5’ AACGGGCCCGATGTT GGTTATAT TTCTTG 3’ were used for PCR amplification.

VIII: Contains amino acids from 2335 to 2767. The primers 5’ GACCAGCTGACAATAATTAATCAAAGTATA 3’ and 5’ AACGGGCCC ATTTTTTTTTTTGTTCAATTC 3’ were used for PCR amplification.

S1.2. PCR primers for PfRH1 deletion constructs designed for pRE4

RII-1: Contains amino acids from 334 to 833. The primers 5’ TCTCGTCAGCTGAAAGATGTAATAAATAATAAG 3’ and 5’ AACGGG CCCatttgttaaattattataacc 3’ were used for PCR amplification.

RII-2: Contains amino acids from 500 to 1000. The primers 5’ GACCAGCTGttacaaatagtacaacaaaaa 3’ and 5’ AACGGGCCCATTTAATATTTTTAAAATTTT 3’ were used for PCR amplification.

RII-3: Contains amino acids from 500 to 833. The primers 5’ GACCAGCTGttacaaatagtacaacaaaaa 3’ and 5’ AACGGGCCC atttgttaaattattataacc 3’ were used for PCR amplification.

S1.3. Universal PCR primers for GFP constructs of PfRH1 (primer [*Xho*I], 5’ CGT ATACTCGAGATGGGGGGGACTGCCGCC 3’; primer [*BamH*I], 5’ CGTATAGGATC CAAGTAAAACAAGGGCTG 3’).

S1.4. PCR primers for recombinant construct expressed in *E.coli*

rRII-3: Contains amino acids from 500 to 833. It was PCR-amplified using primer 5’ GACCATATGttacaaatagtacaacaaaaa 3’ and 5’ AACCTCGAGatttgt taaattattataacc 3’.

rtRVIII: Contains amino acids from 2434 to 2767. It was PCR-amplified using primer 5’ GACGAATTCataaatgaagaagctctacaa 3’ and 5’ AACCTCGAGATTTTTTTT TTTGTTCAATTC 3’.

**S2. Cell Culture and Transfection of COS7 Cells**. COS7 cells (ATCC) were cultured and transfected with pEGFP constructs [1]. The transfected cells were used for erythrocyte-binding assay 40-60 hours post-transfection [2] and observed under an inverted fluorescent microscope (Nikon).

**S3. Enzymatic Treatment of Erythrocytes.** Human erythrocytes were collected in 10% citrate phosphate dextrose and stored at 4°C for up to 4 weeks. The Duffy phenotypes of the erythrocytes were determined by a standard blood banking method [2] and washed 3 times in RPMI-1640 (Invitrogen) for use in erythrocyte-binding assay described below and also in the treatment with neuraminidase, chymotrypsin and trypsin respectively [3].

**S4. Parasites and *in vitro* Culture of *P. falciparum.*** *P. falciparum* clones, W2mef, FCR3, Dd2, 3D7, HB3, T994, and T994ΔRH1 were culture *in vitro* in human erythrocytes from malaria-negative donors and synchronized by standard procedures [4]. The human erythrocytes were treated with neuraminidase (Roche) 1mU/ml and used in the culture of W2mef. After 8 weeks cultivation, W2mef (switched) parasites were selected to grow in sialic acid independent manner as previously described [5].

**S5. Erythrocyte-binding Assay Using rRII-3, rtRVIII, rPvDBPII and Parasite Culture Supernatants.** The recombinant protein was incubated with Duffy-positive human erythrocytes and enzymes treated Duffy-positive human erythrocytes [6]. 500ul culture supernatant was mixed with 100ul packed erythrocytes and enzymes treated erythrocytes as described [7]. The eluted bound protein was separated by SDS-PAGE and detected by Western blotting using a mouse monoclonal anti-penta-histidine (5-His) antibody (Qiagen).

**S6. Amino-Acid Sequence Analysis.** Amino-acid sequence alignments were carried out using BLAST and ClustalW. The coiled-coils prediction was carried out using COILS [8] and then secondary structure predictions were performed [9].

**S7. Animals and Immunization.** Purified His-tag fusion proteins, either rRII-3 or rtRVIII, 50 μg per mouse, was injected into male 7-8 weeks old BALB/C mice (LAC, NUS, Singapore) with Complete Freund’s adjuvant (Pierce) for the first injection and in Incomplete Freund's adjuvant for the subsequent injections in 4 weeks intervals. Blood was drawn from the animals before immunization (pre-immune sera) and two weeks after each boost.

**S8. Erythrocyte Invasion Assay.** Human erythrocytes were treated with neuraminidase at different concentration, 0.01mU/ml, 0.1mU/ml, 1mU/ml, 5mU/ml, 10mU/ml, 20mU/ml, 40mU/ml, 80mU/ml and 100mU/ml. Synchronized late stage schizonts were purified [10] and added in duplicated in a 96 well flat-bottom microtitre plate containing neuraminidase-treated (Nm-treated) erythrocytes and complete RPMI 1640. Nm-treated or normal erythrocytes were to give final parasitaemia of 2% and haematocrit of 4% in a total volumn of 150μl/well. 1,000 to 2,000 erythrocytes were scored for presence of rings on Giemsa-stained smears after 24 h for reinvasion. Invasion was present as % parasitaemia. Parasitaemia (%) = total no. of RBCs infected with rings / total of RBCs × 100. Invasion of Nm-treated erythrocytes was compared with positive control of invasion of the same parasite clones into the normal erythrocytes. Invasion inhibition efficiencies were determined as follows: Inhibition efficiency (%) = (1-Inv(Nm)_conc._) /Inv(positive)) × 100. Data shown are from two separate experiments. Experimental data are presented as the mean ± SE. One way analysis of variance (ANOVA) was used with a post hoc (Bonferroni) test to determine the difference effect of neuraminidase between parasite lines. The significance level was set at *p* < 0.05.

**S9. Recombinant proteins invasion inhibition assay and invasion competition assay.** Synchronized late stage W2mef schizonts were purified [10] and added in triplicates in a 96 well flat-bottom microtitre plate containing 40ul of final concentration of 0.195, 0.39, 0.78, 1.56, 3.12 and 6.25µM of either rRII-3 or rtRVIII. In the competition assay, αrRII-3 at 1:40 dilution was incubated with final concentration of 0.195, 0.39, 0.78, 1.56, 3.12 6.25, 12.5 and 25µM of either rRII-3 or rtRVIII for 90 min at room temperature before adding to the culture. 1,000 to 2,000 erythrocytes were scored for presence of rings on Giemsa-stained smears after 24 h for reinvasion. Invasion was present as (%) parasitaemia. Parasitaemia (%) = total no. of RBCs infected with rings / total of RBCs × 100. Invasion in the presence of protein or protein with αrtRII-3 was compared with positive control of growing into the normal complete RPMI 1640. Invasion inhibition efficiencies were determined as follows: Inhibition efficiency (%) = (1-Inv(protein or protein with αrtRII-3)_conc._) /Inv(positive)) × 100. Experimental data are presented as the mean ± SE.

**S10. Oligomerization state of PfRH1 using size exclusion chromatography.** Five hundred microlitre of recombinant PfRH1-RII-3 at a concentration of 2 mg/ml was subjected to size exclusion chromatography using a pre-packed column of Superdex-200 (HR-10/30) (GE healthcare Amersham, Upsala, Sweden) with 50mM HEPES (pH7.0), 150mM of NaCl, 1mM of DTT and 100mM of L-arginine as the running buffer. The protein was eluted with a partition coefficient (K_av_) of 0.124 (~molecular mass of 222kDa) which corresponds to a multimer of around 5 molecules of recombinant RII-3.

| Marker protein | Molecular mass (kDa) | K_av_ |
| --- | --- | --- |
| Blue dextran | 2000 | 0.104 |
| Myosin | 200 | 0.140 |
| Lactate dehydrogenase | 140 | 0.157 |
| Betagalactosidase | 116 | 0.272 |
| Conalbumin | 75 | 0.423 |
| Chymotrypsinogen | 25 | 0.846 |
| PfRH1 | 222 | 0.124 |

**References**

1. Mayor A, Bir N, Sawhney R, Singh S, Pattnaik P, et al. (2005) Receptor-binding residues lie in central regions of Duffy-binding-like domains involved in red cell invasion and cytoadherence by malaria parasites. Blood 105: 2557-2563.

2. Chitnis CE, Miller LH (1994) Identification of the erythrocyte binding domains of Plasmodium vivax and Plasmodium knowlesi proteins involved in erythrocyte invasion. J Exp Med 180: 497-506.

3. Mayer DC, Mu JB, Kaneko O, Duan J, Su XZ, et al. (2004) Polymorphism in the Plasmodium falciparum erythrocyte-binding ligand JESEBL/EBA-181 alters its receptor specificity. Proc Natl Acad Sci U S A 101: 2518-2523.

4. Trager W, Jensen JB (1976) Human malaria parasites in continuous culture. Science 193: 673-675.

5. Stubbs J, Simpson KM, Triglia T, Plouffe D, Tonkin CJ, et al. (2005) Molecular mechanism for switching of P. falciparum invasion pathways into human erythrocytes. Science 309: 1384-1387.

6. Singh SK, Singh AP, Pandey S, Yazdani SS, Chitnis CE, et al. (2003) Definition of structural elements in Plasmodium vivax and P. knowlesi Duffy-binding domains necessary for erythrocyte invasion. Biochem J 374: 193-198.

7. Triglia T, Thompson J, Caruana SR, Delorenzi M, Speed T, et al. (2001) Identification of proteins from Plasmodium falciparum that are homologous to reticulocyte binding proteins in Plasmodium vivax. Infect Immun 69: 1084-1092.

8. Lupas A, Van Dyke M, Stock J (1991) Predicting coiled coils from protein sequences. Science 252: 1162-1164.

9. Rost B, Liu J (2003) The PredictProtein server. Nucleic Acids Res 31: 3300-3304.

10. Bharara R, Singh S, Pattnaik P, Chitnis CE, Sharma A (2004) Structural analogs of sialic acid interfere with the binding of erythrocyte binding antigen-175 to glycophorin A, an interaction crucial for erythrocyte invasion by Plasmodium falciparum. Mol Biochem Parasitol 138: 123-129.
