# Supplementary material for: Antibodies Targeting the PfRH1 Binding Domain Inhibit Invasion of Plasmodium falciparum Merozoites
Source: PLoS Pathog. 2008 Jul 11;4(7):e1000104. doi: 10.1371/journal.ppat.1000104 (PMC2438614; doi:10.1371/journal.ppat.1000104)
Supplement: Figure S1 — Sequence alignment of putative binding regions of different RH members (0.94 MB DOC) [file ppat.1000104.s003.doc]

**
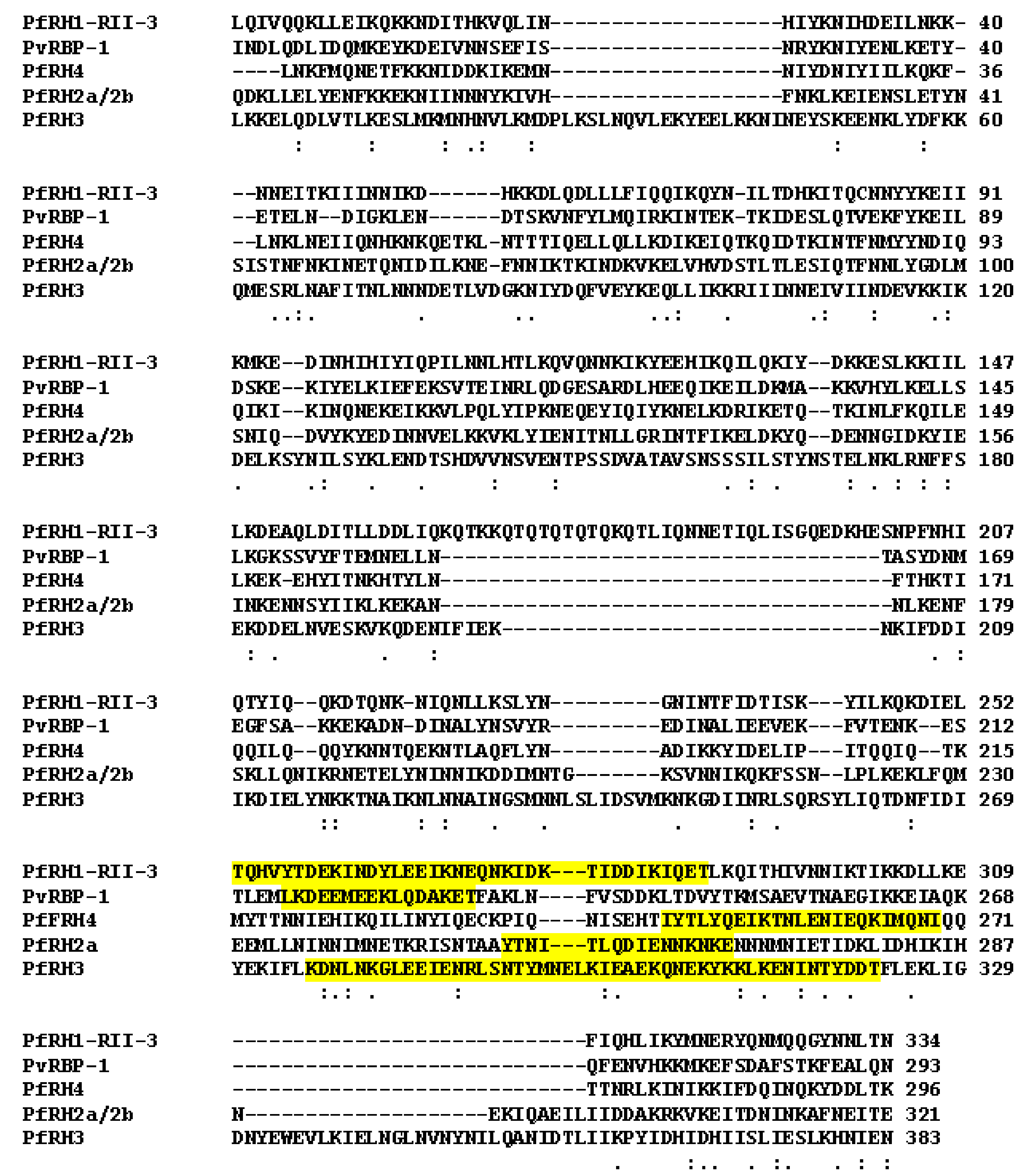
**

Figure S1. Multiple sequence alignment using ClustalW [10] of RH proteins sharing the highest sequence identity with PfRH1-RII-3. PvRBP-1 (Genbank accession no: A42771), PfRH4 (Genbank accession no: AAM47192), PfRH2a (Genbank accession no: AAK19224), PfRH2b (Genbank accession no: AAN39448) and PfRH3 (Genbank accession no: XP_00135906). Pairwise sequence identities are (PfRH1-RII-3, PvRBP-1) 22%, (PfRH1-RII-3, PfRH4) 23%, (PfRH1-RII-3, PfRH2a/2b) 15% and (PfRH1-RII-3, PfRH3) 16%. ":" means that conserved substitutions have been observed and "." means that semi-conserved substitutions are observed. The yellow color boxes mean predicted conserved coiled-coil region in the C-terminal domain which might be involved in protein multimerization.
