# Supplementary material for: Antibodies Targeting the PfRH1 Binding Domain Inhibit Invasion of Plasmodium falciparum Merozoites
Source: PLoS Pathog. 2008 Jul 11;4(7):e1000104. doi: 10.1371/journal.ppat.1000104 (PMC2438614; doi:10.1371/journal.ppat.1000104)
Supplement: Figure S2 — Secondary Structure Prediction of minimal binding region (0.42 MB DOC) [file ppat.1000104.s004.doc]

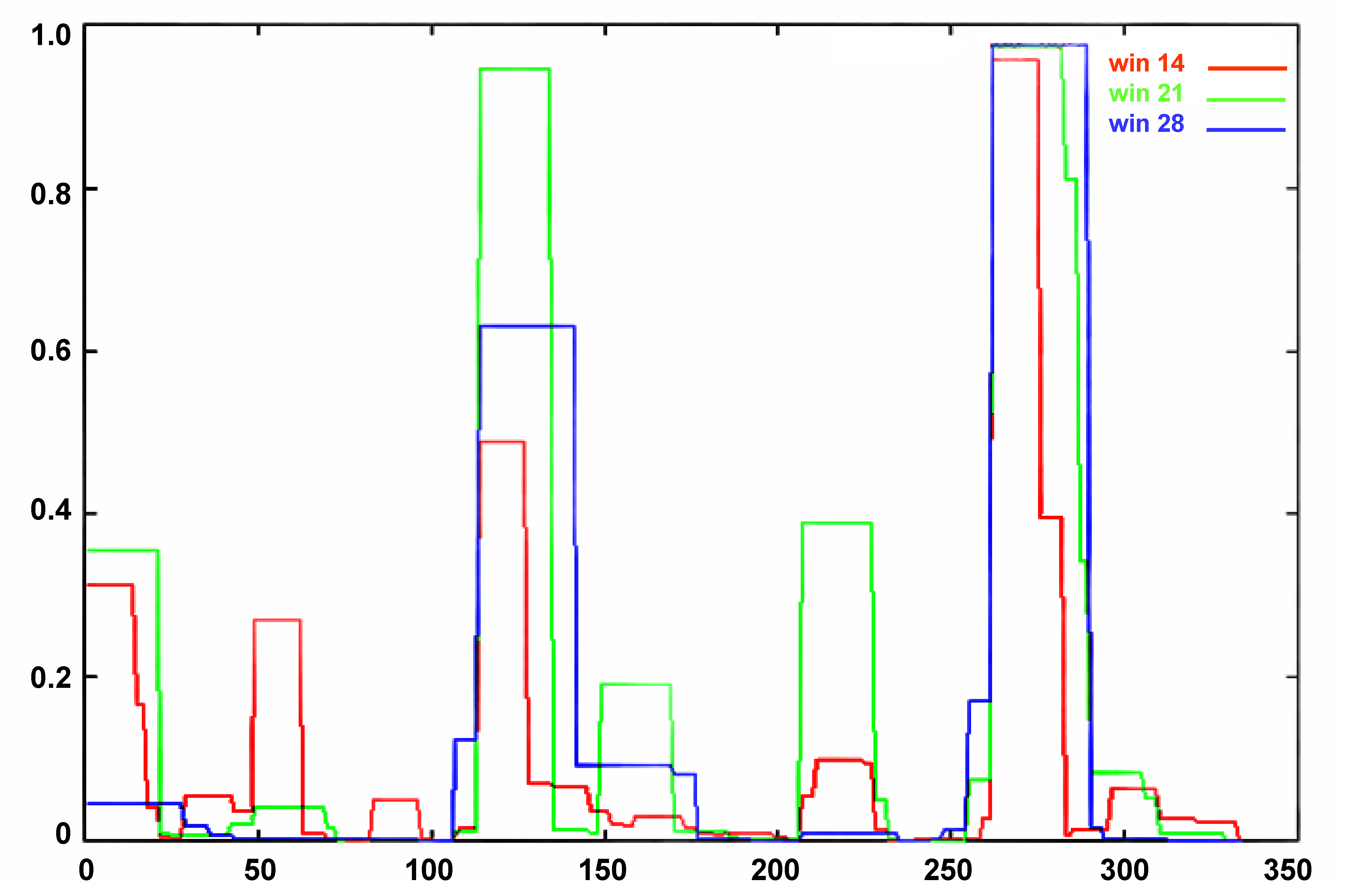


Figure S2. Results of the coiled coils prediction methods for the RII-3 minimal binding protein. The calculation was done using three different window sizes of 14, 21 and 28 amino acids respectively. Even the most stringent criteria (window of 28) reveals an  α-helical coiled coil with a high probability centered at residue 275 within the C-terminal end of the RII-3 sequence.
