# Supplementary material for: Antibodies Targeting the PfRH1 Binding Domain Inhibit Invasion of Plasmodium falciparum Merozoites
Source: PLoS Pathog. 2008 Jul 11;4(7):e1000104. doi: 10.1371/journal.ppat.1000104 (PMC2438614; doi:10.1371/journal.ppat.1000104)
Supplement: Figure S3 — Size exclusion chromatography of recombinant RII-3 (0.52 MB DOC) [file ppat.1000104.s005.doc]

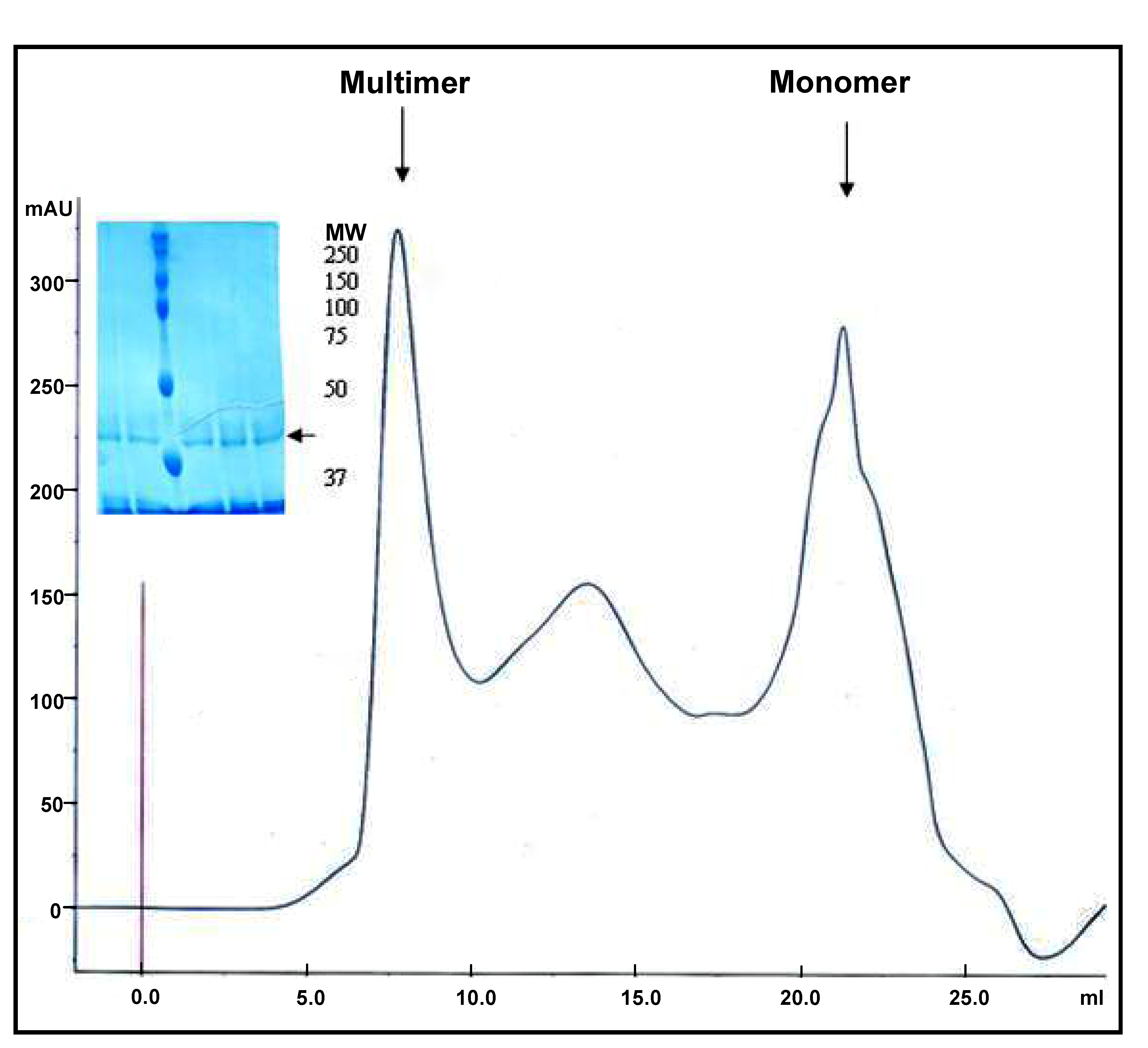


Figure S3. Size exclusion chromatography profile of recombinant RII-3. Elution of protein peaks representing multimer of approximately 222 kDa and monomer of approximately 42 kDa are indicated. Picture insert shows the 12% SDS-PAGE gel of fractions collected during elution of multimeric peak (bracket) eluting with a K_av_ of 0.124.
