# Supplementary material for: Antibodies Targeting the PfRH1 Binding Domain Inhibit Invasion of Plasmodium falciparum Merozoites
Source: PLoS Pathog. 2008 Jul 11;4(7):e1000104. doi: 10.1371/journal.ppat.1000104 (PMC2438614; doi:10.1371/journal.ppat.1000104)
Supplement: Figure S4 — Western blot of T994 and T994ΔRH1 extracts using anti-RH1 antibodies (0.38 MB DOC) [file ppat.1000104.s006.doc]

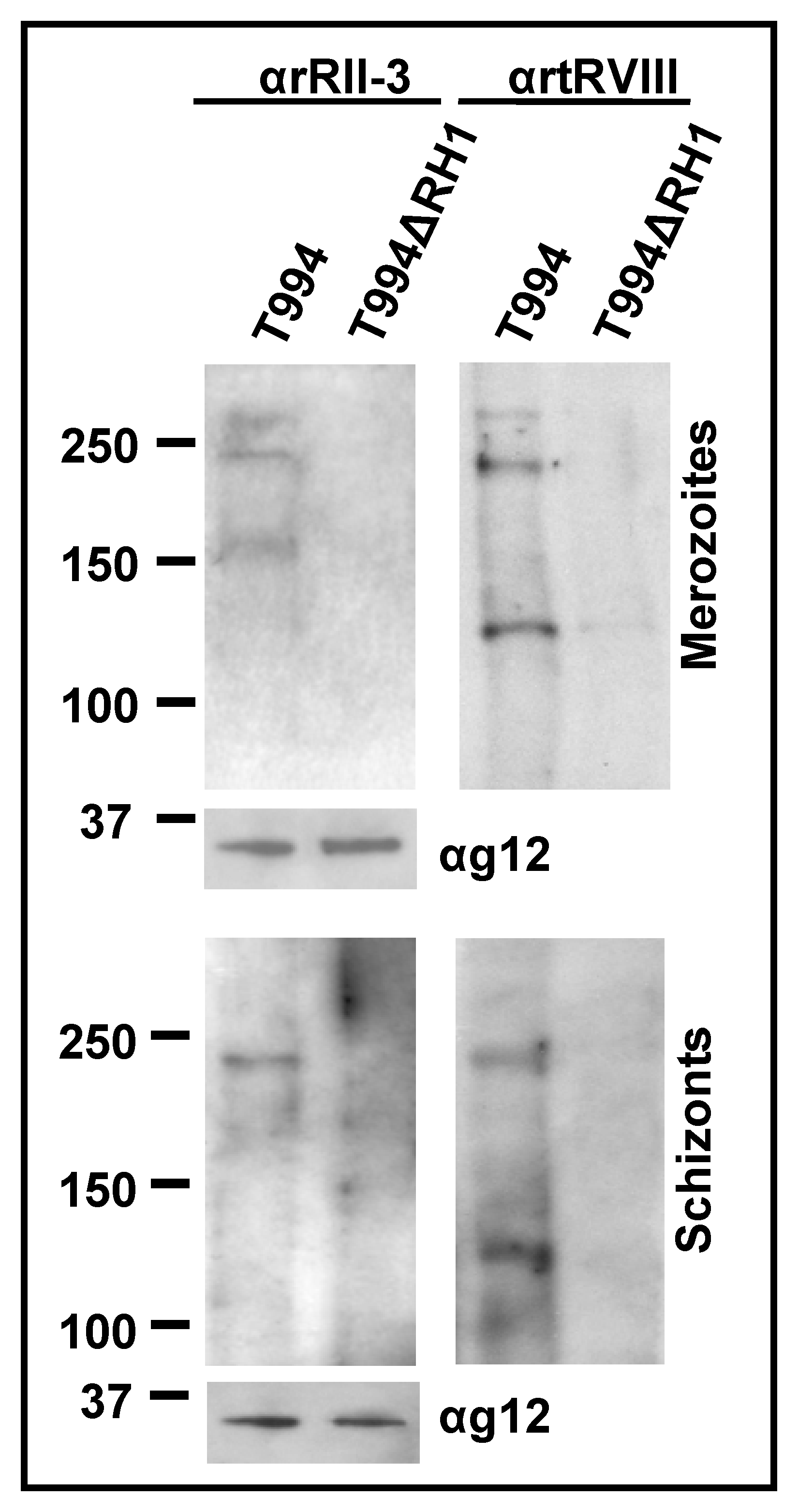


Figure S4. Western analysis of PfRH1 expression on merozoites and late stage schizonts from T994 and T994∆RH1 probed with αrRII-3, αrtRVIII and αg12 as loading control. The expected protein of about 240 kDa was only detected by both antisera in T994 parasites. Molecular sizes are indicated on the left (in kDa).
