# Supplementary material for: Antibodies Targeting the PfRH1 Binding Domain Inhibit Invasion of Plasmodium falciparum Merozoites
Source: PLoS Pathog. 2008 Jul 11;4(7):e1000104. doi: 10.1371/journal.ppat.1000104 (PMC2438614; doi:10.1371/journal.ppat.1000104)
Supplement: Figure S5 — Invasion of T994 and T994ΔRH1 into neuraminidasse-treated erythrocytes (0.32 MB DOC) [file ppat.1000104.s007.doc]

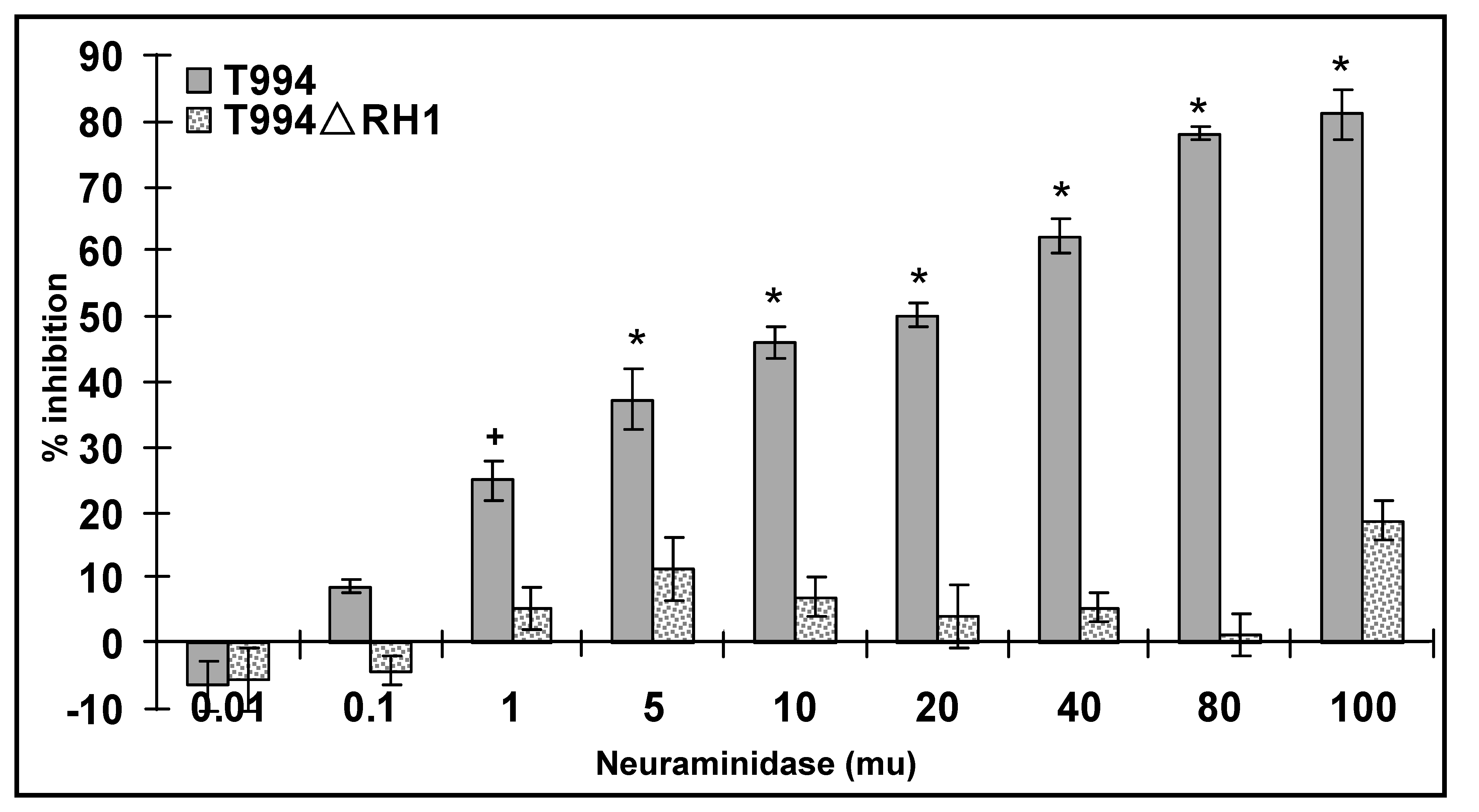


Figure S5. Invasion of T994 and T994∆RH1 into neuraminidasse-treated erythrocytes. Merozoites of T994 parasite are not able to invade Neuraminidasse-treated erythrocytes. The error bar denotes the SE. + *p* < 0.05 and * *p* < 0.001, indicating the significant difference of the sensitivity to neuraminidase between T994 and T994∆RH1.
