# Supplementary material for: Antibodies Targeting the PfRH1 Binding Domain Inhibit Invasion of Plasmodium falciparum Merozoites
Source: PLoS Pathog. 2008 Jul 11;4(7):e1000104. doi: 10.1371/journal.ppat.1000104 (PMC2438614; doi:10.1371/journal.ppat.1000104)
Supplement: Figure S6 — Invasion inhibition assay and invasion competition assay with rRII-3 in W2mef (0.44 MB DOC) [file ppat.1000104.s008.doc]

**
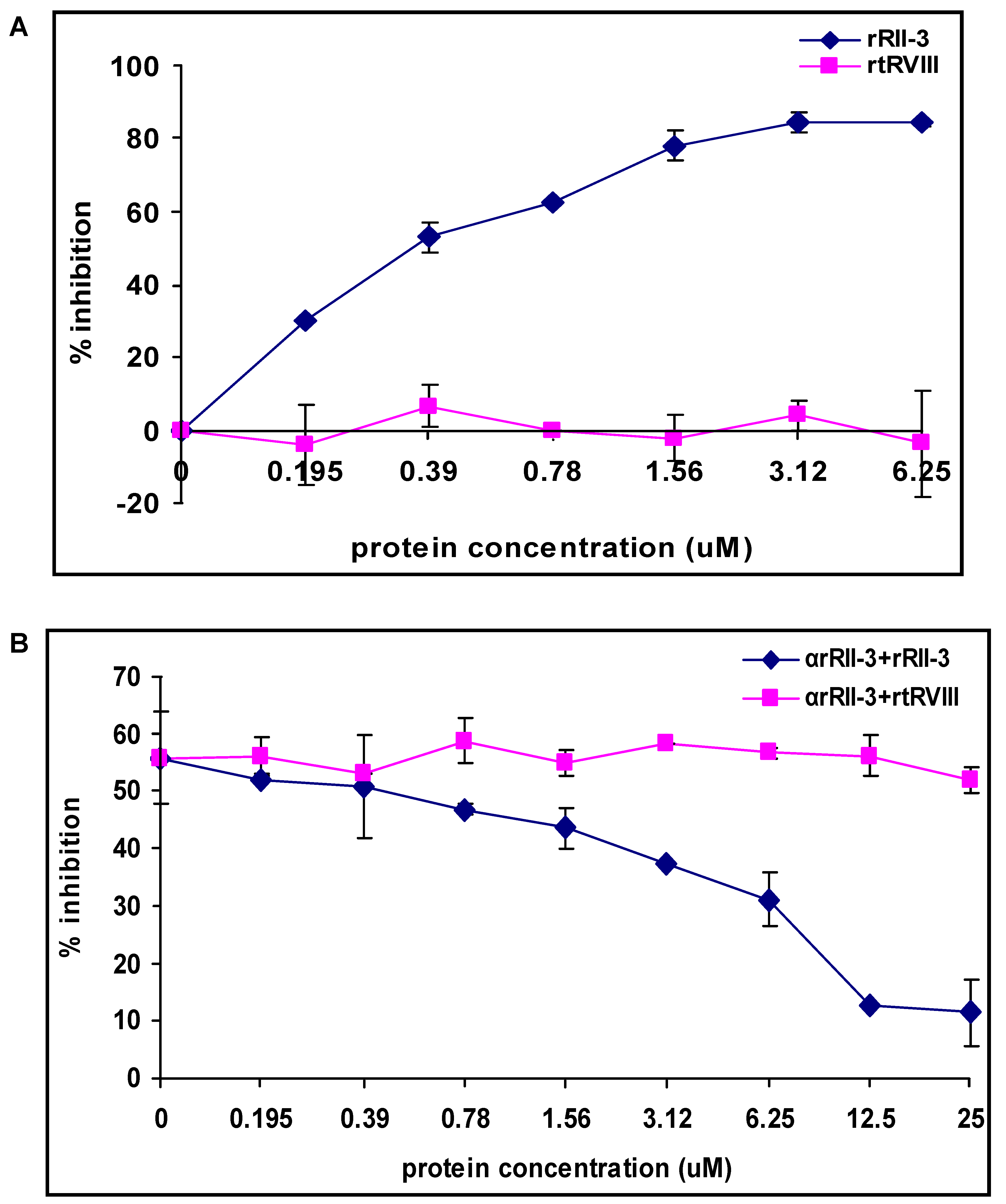
**

Figure S6. Invasion inhibition assay and invasion competition assay with rRII-3 in W2mef. **(A)** The invasion was blocked by rRII-3 not rtRVIII in a concentration-dependent manner with an IC_50_ of 0.39μM. **(B)** Competition assay was carried out after pre-incubating αrRII-3 at 1:40 dilution with either rRII-3 or rtRVIII. Recombinant protein rRII-3 was able to greatly reduce αrRII-3 effects on invasion in a concentration-dependent manner. No inhibition was found by rtRVIII. The error bar denotes the SE.
