# Supplementary material for: Antibodies Targeting the PfRH1 Binding Domain Inhibit Invasion of Plasmodium falciparum Merozoites
Source: PLoS Pathog. 2008 Jul 11;4(7):e1000104. doi: 10.1371/journal.ppat.1000104 (PMC2438614; doi:10.1371/journal.ppat.1000104)
Supplement: Figure S7 — Western blot of merozoite extracts from different parasite lines using anti-RH1 antibodies (0.28 MB DOC) [file ppat.1000104.s009.doc]

**
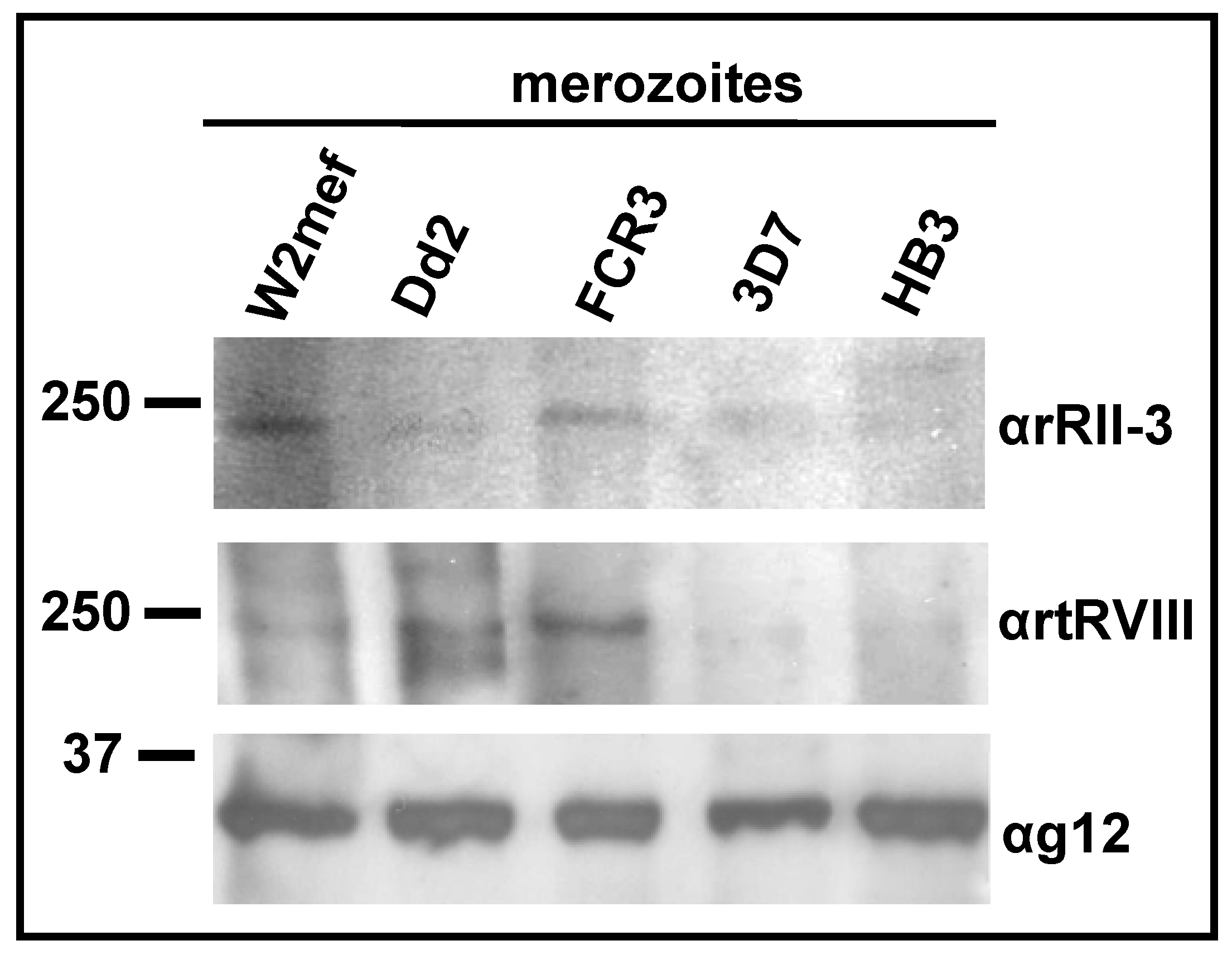
**

Figure S7. Western blot of merozoites from all 5 clones used in this study and probed with αrRII-3, αrtRVIII and αg12. There was much lower RH1 expression in 3D7 and HB3 clones compared with W2mef, Dd2 and FCR3. Molecular sizes are indicated on the left (in kDa).
