# Supplementary material for: Antibodies Targeting the PfRH1 Binding Domain Inhibit Invasion of Plasmodium falciparum Merozoites
Source: PLoS Pathog. 2008 Jul 11;4(7):e1000104. doi: 10.1371/journal.ppat.1000104 (PMC2438614; doi:10.1371/journal.ppat.1000104)
Supplement: Figure S8 — Invasion of W2mef and W2mef (switched) parasites into neuraminidasse-treated erythrocytes (0.40 MB DOC) [file ppat.1000104.s010.doc]

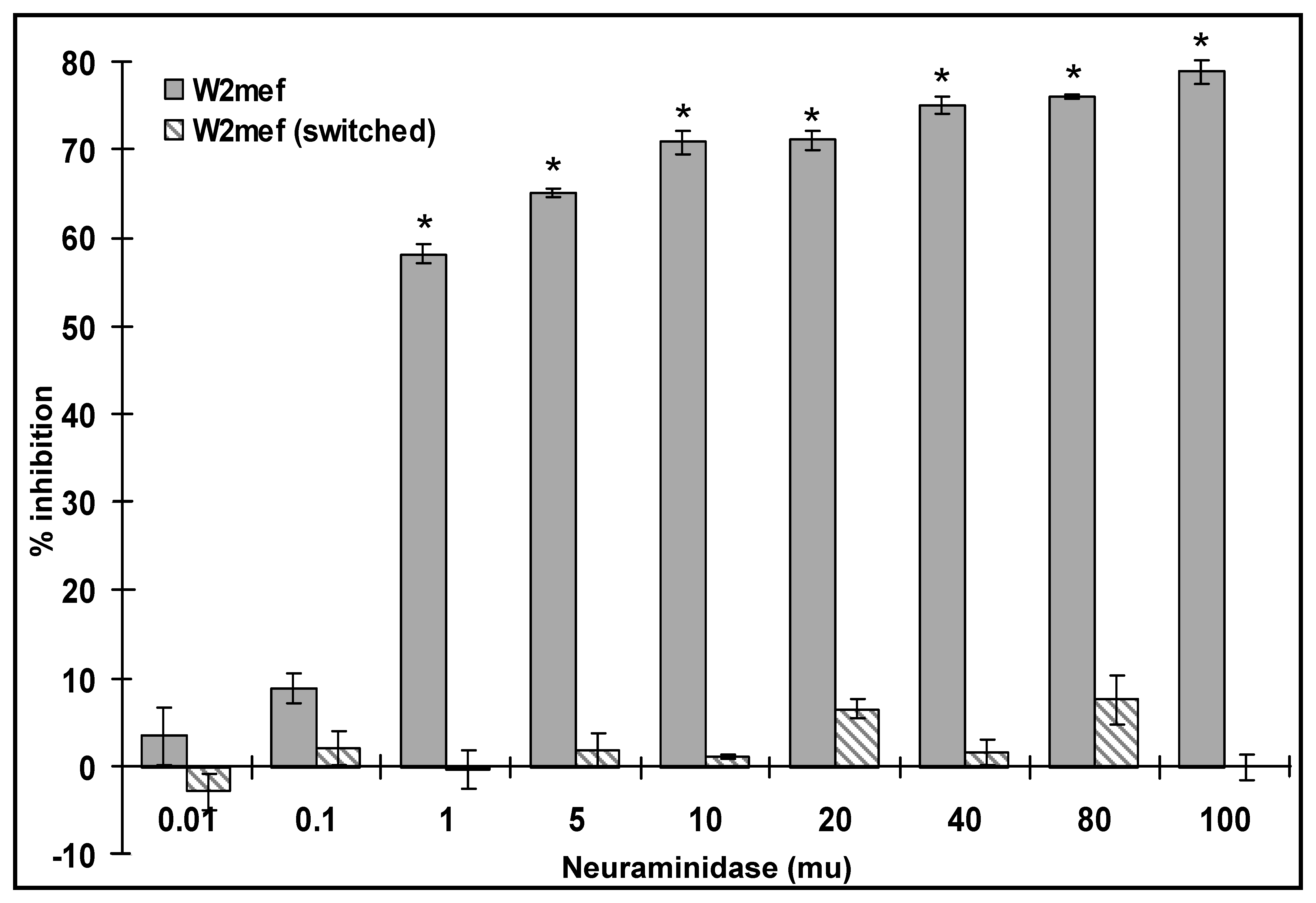


Figure S8. Invasion of W2mef and W2mef (switched) parasites into neuraminidasse-treated erythrocytes. Merozoites of W2mef (switched) parasites are able to invade Neuraminidase-treated erythrocytes successfully. The error bar denotes the SE. * *p* < 0.001 indicating the significant difference of the sensitivity to neuraminidase between W2mef and W2mef (switched) parasites.
