# Supplementary material for: Antibodies Targeting the PfRH1 Binding Domain Inhibit Invasion of Plasmodium falciparum Merozoites
Source: PLoS Pathog. 2008 Jul 11;4(7):e1000104. doi: 10.1371/journal.ppat.1000104 (PMC2438614; doi:10.1371/journal.ppat.1000104)
Supplement: Figure S9 — Western blot of W2mef and W2mef (switched) merozoite and schizont extracts probed with anti-RH1 antibodies (0.19 MB DOC) [file ppat.1000104.s011.doc]

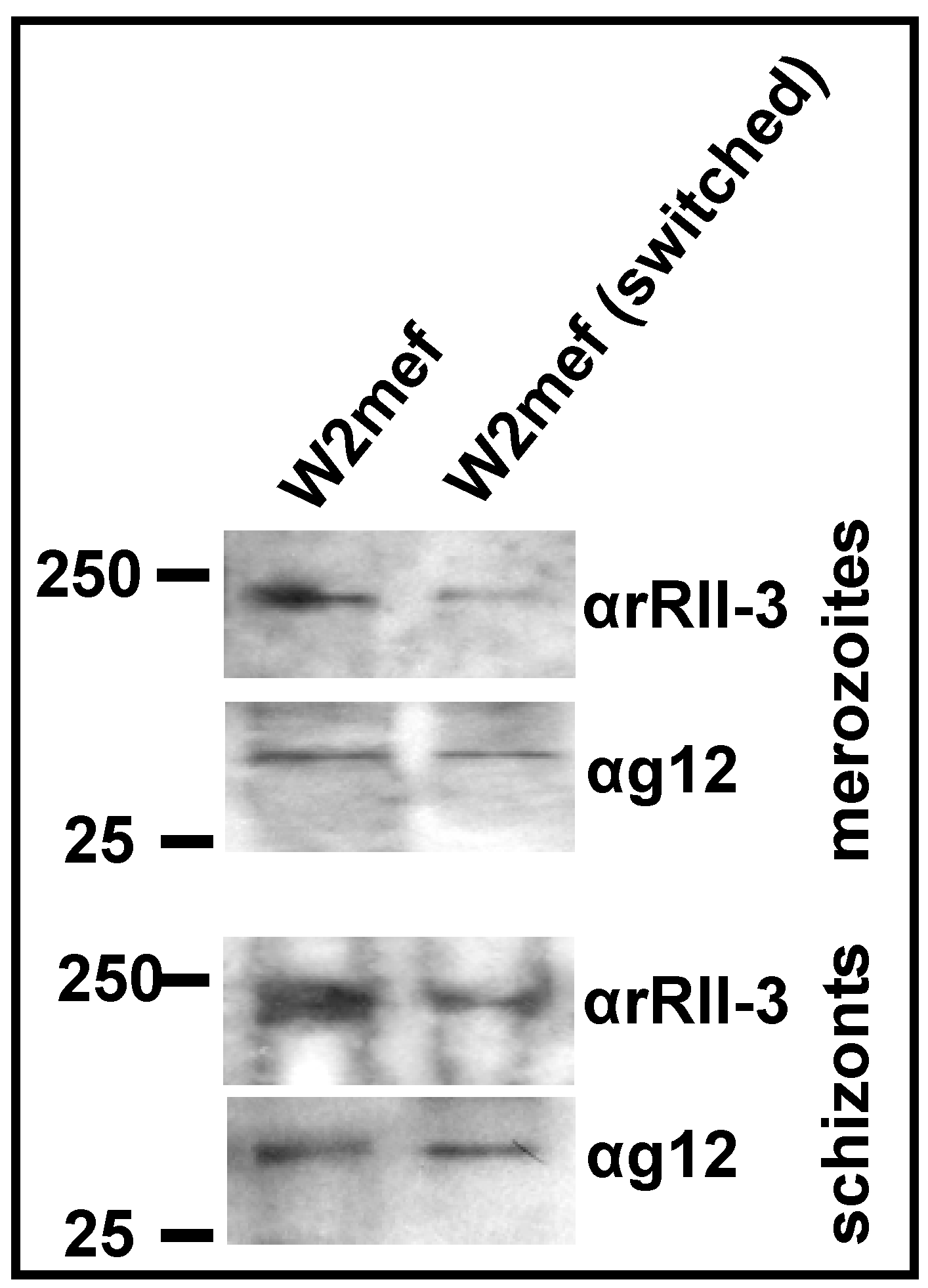


Figure S9. Merozoites and late stage schizonts from W2mef and W2mef (switched) parasite were probed with αrRII-3 and αg12. There was higher decreased in RH1 protein expression level in W2mef (switched) parasites. Molecular sizes are indicated on the left (in kDa).
